# Overuse of medications in low- and middle-income countries: a scoping review

**DOI:** 10.2471/BLT.22.288293

**Published:** 2022-10-31

**Authors:** Loai Albarqouni, Sujeewa Palagama, Julia Chai, Priatharsini Sivananthajothy, Thanya Pathirana, Mina Bakhit, Morteza Arab-Zozani, Respati Ranakusuma, Magnolia Cardona, Anna Scott, Justin Clark, Claire Friedemann Smith, Emmanuel Effa, Eleanor Ochodo, Ray Moynihan, & the Overdiagnosis and Overuse of Healthcare Services in LMICs Network

**Affiliations:** a Institute for Evidence-Based Healthcare, Faculty of Health Sciences and Medicine, Bond University, 14 University Dr, Robina, QLD, 4229, Australia.; b Cumming School of Medicine, University of Calgary, Alberta, Canada.; c School of Medicine and Dentistry, Griffith University, Sunshine Coast, Australia.; d Social Determinants of Health Research Center, Birjand University of Medical Sciences, Birjand, Iran.; e Clinical Epidemiology and Evidence-Based Medicine Unit, Dr Cipto Mangunkusumo Hospital, Jakarta, Indonesia.; f Nuffield Department of Primary Care Health Sciences, University of Oxford, Oxford, England.; g Department of Internal Medicine, University of Calabar, Calabar, Nigeria.; h Centre for Global Health Research, Kenya Medical Research Institute, Nairobi, Kenya.

## Abstract

**Objective:**

To identify and summarize the evidence about the extent of overuse of medications in low- and middle-income countries, its drivers, consequences and potential solutions.

**Methods:**

We conducted a scoping review by searching the databases PubMed®, Embase®, APA PsycINFO® and Global Index Medicus using a combination of MeSH terms and free text words around overuse of medications and overtreatment. We included studies in any language published before 25 October 2021 that reported on the extent of overuse, its drivers, consequences and solutions.

**Findings:**

We screened 3489 unique records and included 367 studies reporting on over 5.1 million prescriptions across 80 low- and middle-income countries – with studies from 58.6% (17/29) of all low-, 62.0% (31/50) of all lower-middle- and 60.0% (33/55) of all upper-middle-income countries. Of the included studies, 307 (83.7%) reported on the extent of overuse of medications, with estimates ranging from 7.3% to 98.2% (interquartile range: 30.2–64.5). Commonly overused classes included antimicrobials, psychotropic drugs, proton pump inhibitors and antihypertensive drugs. Drivers included limited knowledge of harms of overuse, polypharmacy, poor regulation and financial influences. Consequences were patient harm and cost. Only 11.4% (42/367) of studies evaluated solutions, which included regulatory reforms, educational, deprescribing and audit–feedback initiatives.

**Conclusion:**

Growing evidence suggests overuse of medications is widespread within low- and middle-income countries, across multiple drug classes, with few data of solutions from randomized trials. Opportunities exist to build collaborations to rigorously develop and evaluate potential solutions to reduce overuse of medications.

## Résumé

**Objectif:**

Identifier et résumer les données démontrant l'ampleur de la surconsommation de médicaments dans les pays à revenu faible et intermédiaire, mais aussi les causes de cette surconsommation, ses conséquences et les pistes de solution.

**Méthodes:**

Nous avons mené une analyse exploratoire en effectuant une recherche dans les bases de données PubMed®, Embase®, APA PsycINFO® et l'Index Medicus mondial à l'aide d'une combinaison de termes MeSH et de mots en texte libre liés à la surconsommation de médicaments et aux surtraitements. Nous avons inclus des études rédigées dans toutes les langues et publiées avant le 25 octobre 2021, qui mentionnaient l'ampleur de la surconsommation, ses causes, ses conséquences et les pistes de solution.

**Résultats:**

Nous avons passé en revue 3489 documents uniques et conservé 367 études faisant état de plus de 5,1 millions d'ordonnances dans 80 pays à revenu faible et intermédiaire – ces études concernaient 58,6% (17/29) des pays à revenu faible, 62,0 % (31/50) de ceux à revenu intermédiaire de la tranche inférieure et 60,0% (33/55) de ceux à revenu intermédiaire de la tranche supérieure. Sur l'ensemble des études reprises, 307 (83,7%) signalaient l'ampleur de la surconsommation de médicaments, avec des estimations comprises entre 7,3% et 98,2% (écart interquartile: 30,2-64,5). Cette surconsommation s'observait principalement dans des catégories telles que les antimicrobiens, les substances psychotropes, les inhibiteurs de la pompe à protons et les antihypertenseurs. Plusieurs causes étaient évoquées: méconnaissance des dégâts liés à une surconsommation, polypharmacie, mauvaise réglementation et influences économiques. Les conséquences étaient surtout néfastes pour la santé des patients et les coûts engendrés. À peine 11,4% (42/367) des études examinaient des solutions, parmi lesquelles des réformes réglementaires, ainsi que des initiatives de sensibilisation, de déprescription et d'audit–feedback.

**Conclusion:**

Un faisceau croissant de preuves indique que la surconsommation de médicaments est largement répandue dans les pays à revenu faible et intermédiaire, dans de nombreuses catégories de substances, et peu d'informations circulent quant aux solutions issues des essais randomisés. Il existe néanmoins des opportunités de collaboration qui permettraient de développer et d'évaluer rigoureusement des pistes de solution pour lutter contre la surconsommation de médicaments.

## Resumen

**Objetivo:**

Identificar y resumir la evidencia sobre el grado del uso excesivo de medicamentos en los países de ingresos bajos y medios, sus causas, consecuencias y posibles soluciones.

**Métodos:**

Se realizó una revisión exploratoria mediante búsquedas en las bases de datos PubMed®, Embase®, APA PsycINFO® y Global Index Medicus a partir de una combinación de términos MeSH y palabras de texto libre sobre el uso excesivo de medicamentos y el sobretratamiento. Se incluyeron estudios en cualquier idioma publicados antes del 25 de octubre de 2021 y que informaran sobre el grado del uso excesivo, sus causas, consecuencias y soluciones.

**Resultados:**

Se examinaron 3489 registros únicos y se incluyeron 367 estudios que informaron sobre más de 5,1 millones de recetas en 80 países de ingresos bajos y medios, con estudios del 58,6 % (17/29) de todos los países de ingresos bajos, el 62,0 % (31/50) de todos los países de ingresos medios bajos y el 60,0 % (33/55) de los países de ingresos medios altos. De los estudios incluidos, 307 (83,7 %) informaron sobre el grado de uso excesivo de medicamentos, con estimaciones que oscilaban entre el 7,3 % y el 98,2 % (rango intercuartil: 30,2-64,5). Las clases más usadas en exceso incluían los antimicrobianos, los psicofármacos, los inhibidores de la bomba de protones y los antihipertensores. Los factores causantes incluyeron el conocimiento limitado de los daños del uso excesivo, la polifarmacia, la falta de regulación y las influencias financieras. Las consecuencias incluían el daño a los pacientes y el coste. Solo el 11,4 % (42/367) de los estudios evaluaron las soluciones, entre las que se encontraban las reformas normativas, las iniciativas de educación, de desprescripción y de retroalimentación de auditorías.

**Conclusión:**

Cada vez hay más evidencias de que el uso excesivo de medicamentos está muy extendido en los países de ingresos bajos y medios, en múltiples clases de medicamentos, y se dispone de datos insuficientes sobre soluciones procedentes de ensayos aleatorizados. Hay oportunidades de crear colaboraciones para desarrollar y evaluar con rigor posibles soluciones para reducir el uso excesivo de los medicamentos.

## ملخص

الغرض تحديد وتلخيص الأدلة على مدى الاستخدام المفرط للأدوية في الدول ذات الدخل المنخفض والمتوسط، ودوافع هذا الاستخدام، وعواقبه، والحلول الممكنة له.

الطريقة لقد أجرينا مراجعة عن كثب عن طريق البحث في قواعد البيانات PubMed®‎، وEmbase®‎، وAPA PsycINFO®‎، وGlobal Index Medicus باستخدام مجموعة من مصطلحات MeSH، وكلمات نصية حرة بخصوص الاستخدام المفرط للأدوية والمعالجة المفرطة. وقمنا بتضمين دراسات ‎‎‎نُشرت بأية لغة قبل 25 أكتوبر/تشرين أول 2021، وكنا نُبلغ عن مدى الاستخدام المفرط ودوافعه، وعواقبه، وحلوله.

النتائج قمنا بفحص 3489 سجلاً فريداً، وتضمين 367 دراسة كشفت عن أكثر من 5.1 مليون وصفة طبية في 80 دولة ذات دخل منخفض إلى متوسط - مع دراسات من %58.6 (17/29) من كل الدول ذات الدخل المنخفض، و%62.0 (31/50) من كل الدول ذات الدخل المتوسط الأدنى، و%60.0 (33/55) من الدول ذات الدخل المتوسط الأعلى. من بين الدراسات المشمولة، كشفت 307 (%83.7) عن مدى الاستخدام المفرط للأدوية، مع تقديرات تتراوح من %7.3 إلى %98.2 (المدى بين الشرائح الربعية: 30.2 إلى 64.5). وشملت الفئات الأكثر استخدامًا بشكل شائع مضادات الميكروبات، والعقاقير المؤثرة على العقل، ومثبطات مضخة البروتون، والأدوية الخافضة للضغط. تضمنت الدوافع معرفة محدودة بأضرار الاستخدام المفرط، وتعدد الأدوية، وسوء التنظيم، والتأثيرات المالية. كانت العواقب هي حدوث الضرر للمرضى، فضلًا عن التكلفة. قامت %11.4 فقط (42/367) من الدراسات بتقييم الحلول، والتي تضمنت الإصلاحات التنظيمية، والمبادرات التعليمية، ومبادرات الحد من الوصفات الطبية، ومبادرات التدقيق والمراجعة.

الاستنتاج تشير الدلائل المتزايدة إلى أن الاستخدام المفرط للأدوية هو ظاهرة منتشرة في الدول ذات الدخل المنخفض والدخل المتوسط، عبر فئات عديدة من الأدوية، مع بيانات قليلة عن الحلول من التجارب العشوائية. توجد فرص لبناء علاقات تعاون لتطوير، وتقييم الحلول الممكنة بشكل فعال للحد من الاستخدام المفرط للأدوية.

## 摘要

**目的:**

旨在确定和概述关于中低收入国家药物滥用的程度、其驱动因素、后果和潜在解决方案的证据。

**方法:**

我们通过搜索 PubMed®、Embase®、APA PsycINFO® 和 Global Index Medicus 数据库,结合使用 MeSH 主题词和自由词,对药物滥用和过度治疗情况进行了一项范围审查。我们纳入了 2021 年 10 月 25 日之前以任何语言发表的研究,并报告了药物滥用的程度、其驱动因素、结果和解决方案。

**结果:**

我们筛选了 3489 项独特记录,纳入了 367 份研究,报告涉及 80 个中低收入国家的 510 多万份处方,其中所有低收入国家的研究占 58.6% (17/29)、所有中下收入国家的研究占 62.0% (31/50) 以及中上收入国家的研究占 60.0% (33/55)。纳入的研究中,307 (83.7%) 份研究报告了药物滥用的程度,估计范围为 7.3% 至 98.2%(四分位距:30.2–64.5)。常见的滥用药物包括抗菌药物、精神治疗药物、质子泵抑制剂和抗高血压药。驱动因素包括对药物滥用危害的认识有限、多重用药、监管不力以及财务影响。后果是患者受到伤害并承担费用。只有 11.4% (42/367) 研究评估了解决方案,其中包括监管改革、教育、取消处方和审查反馈方案。

**结论:**

越来越多的证据表明中低收入国家普遍存在药物滥用情况,涉及多种药物类别,但有关随机试验中得出的解决方案的数据却很少。应创建机会建立合作,严格开发和评估潜在解决方案,以减少药物滥用情况。

## Резюме

**Цель:**

Определить и обобщить данные о масштабах чрезмерного использования лекарственных средств в странах с низким и средним уровнем дохода, его движущих факторах, последствиях и возможных способах решения проблемы.

**Методы:**

Авторы провели предварительный обзор путем поиска данных в базах PubMed®, Embase®, APA PsycINFO® и Global Index Medicus, используя комбинацию терминов MeSH и слов произвольного текста, касающихся чрезмерного употребления лекарственных средств и передозировки ими. Были включены исследования на всех языках, опубликованные до 25 октября 2021 г., в которых сообщалось о масштабах чрезмерного употребления, его причинах, последствиях и способах решения проблемы.

**Результаты:**

Авторы проанализировали 3489 уникальных записей и включили 367 исследований, в которых сообщалось о более чем 5,1 млн рецептов в 80 странах с низким и средним уровнем дохода. Среди них были исследования из 58,6% (17/29) всех стран с низким уровнем дохода, 62,0% (31/50) всех стран с уровнем дохода ниже среднего и 60,0% (33/55) стран с уровнем дохода выше среднего. Из включенных исследований в 307 (83,7%) сообщалось о степени чрезмерного использования лекарственных средств, при этом оценки колебались от 7,3 до 98,2% (межквартильный диапазон: 30,2–64,5). Как правило, к числу классов чрезмерно используемых лекарственных средств относятся: противомикробные препараты, психотропные средства, ингибиторы протонной помпы и антигипертензивные препараты. К движущим факторам относятся: ограниченные знания о вреде чрезмерного использования, полипрагмазия, слабое регулирование и финансовое влияние. Последствия для пациентов заключаются во вреде, причиненном их здоровью, и дополнительных затратах. Только в 11,4% (42/367) исследований оценивались способы решения проблемы, которые включали программу по реформированию нормативно-правовой базы, инициативы в области образования, а также в направлении постепенного прекращения использования определенных лекарственных средств и внедрения аудита с обратной связью.

**Вывод:**

Все больше данных свидетельствуют о том, что чрезмерное использование лекарственных средств широко распространено в странах с низким и средним уровнем дохода в отношении нескольких классов лекарств, при этом данных о способах решения проблемы, полученных в ходе рандомизированных исследований, немного. Существуют возможности для налаживания сотрудничества с целью тщательной разработки и оценки потенциальных способов сокращения чрезмерного использования лекарственных средств.

## Introduction

Overuse in health care is broadly defined as tests or treatments that are inappropriate, unnecessary or of low value and are likely to cause people more harm than benefit. Hence, health-care overuse is a recognized threat to both human health and health system sustainability.^1^^,^^2^

Estimates of global overuse suggest that 20–40% of health-care resources may be wasted and that these resources might be better invested tackling unmet need, including underuse.^3^^–^^5^ While much of the evidence for overuse arises from high-income countries, where there is greater access to care, the consequences due to overuse may be even more serious in low-resource settings.^1^ For example, medication overuse, a key component of health-care overuse, can threaten both the viability of public budgets, including the universal health coverage, and population health in low- and middle-income countries.^1^

Global initiatives such as Choosing Wisely^6^ and Preventing Overdiagnosis^7^ are increasingly interested in addressing the problem of overuse in low- and middle-income settings. To identify gaps in the evidence-base and develop an agenda for future research and actions, we conducted a scoping review to characterize the evidence about the extent of overuse of medications in these countries, its drivers, consequences and potential solutions to reduce it in low- and middle-income countries. The work has also contributed to building a new global network to take the new agenda forward.

## Methods

We conducted this scoping review following the Joanna Briggs Institute guidance,^8^ using an accelerated approach,^9^ and reported it following the Preferred Reporting Items for Systematic Reviews and Meta-Analyses Extension for Scoping Reviews.^10^ The protocol was prospectively developed and registered at Open Science Framework.^11^

### Search strategy

We searched four electronic databases: PubMed®, Embase®, APA PsycINFO® and Global Index Medicus, from inception until 25 October 2021, using a low- and middle-country search filter from Cochrane^12^ and without language restrictions. We used a combination of MeSH terms and free text words for the following general concepts: overuse of medications/overtreatment (Box 1; available at: https://www.who.int/publications/journals/bulletin/). We searched reference lists of all included studies, and contacted experts in the field to identify relevant and important grey literature.

Box 1 **Search strategy used to identify studies on overuse of medications in low- and middle-income countries**

Search on 21 October 2021

**PubMed®**

(“Medical Overuse”[Mesh] OR Overmedicalization[tiab] OR Overmedicalisation[tiab] OR Overtreatment[tiab] OR “Over-treatment”[tiab] OR ((Overuse[tiab] OR Unnecessary[ti] OR Unwarranted[tiab] OR Inappropriate[ti] OR Deprescribing[tiab] OR De-implementation[tiab] OR Deimplementation[tiab]) AND (Medication[tiab] OR Therapeutic[tiab] OR Therapeutics[tiab] OR Antibiotics[tiab] OR Medicine[ti] OR Medicines[tiab] OR Prescriptions[tiab] OR “Pharmacological treatment”[tiab] OR “Pharmacological treatments”[tiab])))

AND

(afghanistan[Text Word] OR albania[Text Word] OR algeria[Text Word] OR american samoa[Text Word] OR angola[Text Word] OR antigua[Text Word] OR barbuda[Text Word] OR argentina[Text Word] OR armenia[Text Word] OR armenian[Text Word] OR aruba[Text Word] OR azerbaijan[Text Word] OR bahrain[Text Word] OR bangladesh[Text Word] OR barbados[Text Word] OR belarus[Text Word] OR byelarus[Text Word] OR belorussia[Text Word] OR byelorussian[Text Word] OR belize[Text Word] OR british honduras[Text Word] OR benin[Text Word] OR dahomey[Text Word] OR bhutan[Text Word] OR bolivia[Text Word] OR bosnia[Text Word] OR herzegovina[Text Word] OR botswana[Text Word] OR bechuanaland[Text Word] OR brazil[Text Word] OR brasil[Text Word] OR bulgaria[Text Word] OR burkina faso[Text Word] OR burkina fasso[Text Word] OR upper volta[Text Word] OR burundi[Text Word] OR urundi[Text Word] OR cabo verde[Text Word] OR cape verde[Text Word] OR cambodia[Text Word] OR kampuchea[Text Word] OR khmer republic[Text Word] OR cameroon[Text Word] OR cameron[Text Word] OR cameroun[Text Word] OR central african republic[Text Word] OR ubangi shari[Text Word] OR chad[Text Word] OR chile[Text Word] OR china[Text Word] OR colombia[Text Word] OR comoros[Text Word] OR comoro islands[Text Word] OR mayotte[Text Word] OR congo[Text Word] OR zaire[Text Word] OR costa rica[Text Word] OR cote d’ivoire[Text Word] OR cote d’ ivoire[Text Word] OR cote divoire[Text Word] OR cote d ivoire[Text Word] OR Côte d'Ivoire[Text Word] OR croatia[Text Word] OR cuba[Text Word] OR cyprus[Text Word] OR czech republic[Text Word] OR czechoslovakia[Text Word] OR djibouti[Text Word] OR french somaliland[Text Word] OR dominica[Text Word] OR dominican republic[Text Word] OR ecuador[Text Word] OR egypt[Text Word] OR united arab republic[Text Word] OR el salvador[Text Word] OR equatorial guinea[Text Word] OR spanish guinea[Text Word] OR eritrea[Text Word] OR estonia[Text Word] OR eswatini[Text Word] OR swaziland[Text Word] OR ethiopia[Text Word] OR fiji[Text Word] OR gabon[Text Word] OR gabonese republic[Text Word] OR gambia[Text Word] OR georgia[Text Word] OR georgian[Text Word] OR ghana[Text Word] OR gold coast[Text Word] OR gibraltar[Text Word] OR greece[Text Word] OR grenada[Text Word] OR guam[Text Word] OR guatemala[Text Word] OR guinea[Text Word] OR guyana[Text Word] OR guiana[Text Word] OR haiti[Text Word] OR hispaniola[Text Word] OR honduras[Text Word] OR hungary[Text Word] OR india[Text Word] OR indonesia[Text Word] OR timor[Text Word] OR iran[Text Word] OR iraq[Text Word] OR isle of man[Text Word] OR jamaica[Text Word] OR jordan[Text Word] OR kazakhstan[Text Word] OR kazakh[Text Word] OR kenya[Text Word] OR korea[Text Word] OR kosovo[Text Word] OR kyrgyzstan[Text Word] OR kirghizia[Text Word] OR kirgizstan[Text Word] OR kyrgyz republic[Text Word] OR kirghiz[Text Word] OR laos[Text Word] OR lao pdr[Text Word] OR lao people's democratic republic[Text Word] OR latvia[Text Word] OR lebanon[Text Word] OR lesotho[Text Word] OR basutoland[Text Word] OR liberia[Text Word] OR libya[Text Word] OR libyan arab jamahiriya[Text Word] OR lithuania[Text Word] OR macau[Text Word] OR macao[Text Word] OR macedonia[Text Word] OR madagascar[Text Word] OR malagasy republic[Text Word] OR malawi[Text Word] OR nyasaland[Text Word] OR malaysia[Text Word] OR maldives[Text Word] OR indian ocean[Text Word] OR mali[Text Word] OR malta[Text Word] OR micronesia[Text Word] OR kiribati[Text Word] OR marshall islands[Text Word] OR nauru[Text Word] OR northern mariana islands[Text Word] OR palau[Text Word] OR tuvalu[Text Word] OR mauritania[Text Word] OR mauritius[Text Word] OR mexico[Text Word] OR moldova[Text Word] OR moldovian[Text Word] OR mongolia[Text Word] OR montenegro[Text Word] OR morocco[Text Word] OR ifni[Text Word] OR mozambique[Text Word] OR portuguese east africa[Text Word] OR myanmar[Text Word] OR burma[Text Word] OR namibia[Text Word] OR nepal[Text Word] OR netherlands antilles[Text Word] OR nicaragua[Text Word] OR niger[Text Word] OR nigeria[Text Word] OR oman[Text Word] OR muscat[Text Word] OR pakistan[Text Word] OR panama[Text Word] OR papua new guinea[Text Word] OR paraguay[Text Word] OR peru[Text Word] OR philippines[Text Word] OR philipines[Text Word] OR phillipines[Text Word] OR phillippines[Text Word] OR poland[Text Word] OR polish people's republic[Text Word] OR portugal[Text Word] OR portuguese republic[Text Word] OR puerto rico[Text Word] OR romania[Text Word] OR russia[Text Word] OR russian federation[Text Word] OR ussr[Text Word] OR soviet union[Text Word] OR union of soviet socialist republics[Text Word] OR rwanda[Text Word] OR ruanda[Text Word] OR samoa[Text Word] OR pacific islands[Text Word] OR polynesia[Text Word] OR samoan islands[Text Word] OR sao tome and principe[Text Word] OR saudi arabia[Text Word] OR senegal[Text Word] OR serbia[Text Word] OR seychelles[Text Word] OR sierra leone[Text Word] OR slovakia[Text Word] OR slovak republic[Text Word] OR slovenia[Text Word] OR melanesia[Text Word] OR solomon island[Text Word] OR solomon islands[Text Word] OR norfolk island[Text Word] OR somalia[Text Word] OR south africa[Text Word] OR south sudan[Text Word] OR sri lanka[Text Word] OR ceylon[Text Word] OR saint kitts and nevis[Text Word] OR st kitts and nevis[Text Word] OR saint lucia[Text Word] OR st lucia[Text Word] OR saint vincent[Text Word] OR st vincent[Text Word] OR grenadines[Text Word] OR sudan[Text Word] OR suriname[Text Word] OR surinam[Text Word] OR Syrian Arab Republic[Text Word] OR syrian arab republic[Text Word] OR tajikistan[Text Word] OR tadjikistan[Text Word] OR tadzhikistan[Text Word] OR tadzhik[Text Word] OR tanzania[Text Word] OR tanganyika[Text Word] OR thailand[Text Word] OR siam[Text Word] OR timor leste[Text Word] OR east timor[Text Word] OR togo[Text Word] OR togolese republic[Text Word] OR tonga[Text Word] OR trinidad[Text Word] OR tobago[Text Word] OR tunisia[Text Word] OR turkey[Text Word] OR turkmenistan[Text Word] OR turkmen[Text Word] OR uganda[Text Word] OR ukraine[Text Word] OR uruguay[Text Word] OR uzbekistan[Text Word] OR uzbek[Text Word] OR vanuatu[Text Word] OR new hebrides[Text Word] OR venezuela[Text Word] OR vietnam[Text Word] OR viet nam[Text Word] OR middle east[Text Word] OR west bank[Text Word] OR gaza[Text Word] OR palestine[Text Word] OR yemen[Text Word] OR yugoslavia[Text Word] OR zambia[Text Word] OR zimbabwe[Text Word] OR northern rhodesia[Text Word] OR global south[Text Word] OR africa south of the sahara[Text Word] OR sub-Saharan africa[Text Word] OR sub-Saharan africa[Text Word] OR central africa[Text Word] OR north africa[Text Word] OR northern africa[Text Word] OR magreb[Text Word] OR maghrib[Text Word] OR sahara[Text Word] OR southern africa[Text Word] OR east africa[Text Word] OR eastern africa[Text Word] OR west africa[Text Word] OR western africa[Text Word] OR west indies[Text Word] OR indian ocean islands[Text Word] OR caribbean[Text Word] OR central america[Text Word] OR latin america[Text Word] OR south america[Text Word] OR central asia[Text Word] OR north asia[Text Word] OR northern asia[Text Word] OR southeastern asia[Text Word] OR south eastern asia[Text Word] OR south-east asia[Text Word] OR south-east asia[Text Word] OR western asia[Text Word] OR east europe[Text Word] OR eastern europe[Text Word] OR developing country[Text Word] OR developing countries[Text Word] OR developing nation[Text Word] OR developing nations[Text Word] OR developing population[Text Word] OR developing populations[Text Word] OR developing world[Text Word] OR less developed country[Text Word] OR less developed countries[Text Word] OR less developed nation[Text Word] OR less developed nations[Text Word] OR less developed world[Text Word] OR lesser developed countries[Text Word] OR lesser developed nations[Text Word] OR under developed country[Text Word] OR under developed countries[Text Word] OR under developed nations[Text Word] OR under developed world[Text Word] OR underdeveloped country[Text Word] OR underdeveloped countries[Text Word] OR underdeveloped nation[Text Word] OR underdeveloped nations[Text Word] OR underdeveloped population[Text Word] OR underdeveloped populations[Text Word] OR underdeveloped world[Text Word] OR middle income country[Text Word] OR middle income countries[Text Word] OR middle income nation[Text Word] OR middle income nations[Text Word] OR middle income population[Text Word] OR middle income populations[Text Word] OR low income country[Text Word] OR low income countries[Text Word] OR low income nation[Text Word] OR low income nations[Text Word] OR low income population[Text Word] OR low income populations[Text Word] OR lower income country[Text Word] OR lower income countries[Text Word] OR lower income nations[Text Word] OR lower income population[Text Word] OR lower income populations[Text Word] OR underserved countries[Text Word] OR underserved nations[Text Word] OR underserved population[Text Word] OR underserved populations[Text Word] OR under served population[Text Word] OR under served populations[Text Word] OR deprived countries[Text Word] OR deprived population[Text Word] OR deprived populations[Text Word] OR poor country[Text Word] OR poor countries[Text Word] OR poor nation[Text Word] OR poor nations[Text Word] OR poor population[Text Word] OR poor populations[Text Word] OR poor world[Text Word] OR poorer countries[Text Word] OR poorer nations[Text Word] OR poorer population[Text Word] OR poorer populations[Text Word] OR developing economy[Text Word] OR developing economies[Text Word] OR less developed economy[Text Word] OR less developed economies[Text Word] OR underdeveloped economies[Text Word] OR middle income economy[Text Word] OR middle income economies[Text Word] OR low income economy[Text Word] OR low income economies[Text Word] OR lower income economies[Text Word] OR low gdp[Text Word] OR low gnp[Text Word] OR low gross domestic[Text Word] OR low gross national[Text Word] OR lower gdp[Text Word] OR lower gross domestic[Text Word] OR lmic[Text Word] OR lmics[Text Word] OR third world[Text Word] OR lami country[Text Word] OR lami countries[Text Word] OR transitional country[Text Word] OR transitional countries[Text Word] OR emerging economies[Text Word] OR emerging nation[Text Word] OR emerging nations[Text Word])

**Embase®**

(“Unnecessary Procedure”/exp/mj OR Overmedicalization:ti,ab OR Overmedicalisation:ti,ab OR Overtreatment:ti,ab OR Over-treatment:ti,ab OR ((Overuse:ti,ab OR Unnecessary:ti OR Unwarranted:ti,ab OR Inappropriate:ti OR Deprescribing:ti,ab OR De-implementation:ti,ab OR Deimplementation:ti,ab) AND (Medication:ti,ab OR Therapeutic:ti,ab OR Therapeutics:ti,ab OR Antibiotics:ti,ab OR Medicine:ti OR Medicines:ti,ab OR Prescriptions:ti,ab OR “Pharmacological treatment”:ti,ab OR “Pharmacological treatments”:ti,ab)))

AND

(afghanistan OR albania OR algeria OR “american samoa” OR angola OR antigua OR barbuda OR argentina OR armenia OR armenian OR aruba OR azerbaijan OR bahrain OR bangladesh OR barbados OR belarus OR byelarus OR belorussia OR byelorussian OR belize OR “british honduras” OR benin OR dahomey OR bhutan OR bolivia OR bosnia OR herzegovina OR botswana OR bechuanaland OR brazil OR brasil OR bulgaria OR “burkina faso” OR “burkina fasso” OR “upper volta” OR burundi OR urundi OR “cabo verde” OR “cape verde” OR cambodia OR kampuchea OR “khmer republic” OR cameroon OR cameron OR cameroun OR “central african republic” OR “ubangi shari” OR chad OR chile OR china OR colombia OR comoros OR “comoro islands” OR mayotte OR congo OR zaire OR “costa rica” OR “cote divoire” OR “cote d ivoire” OR “cote divoire” OR “cote d ivoire” OR “ivory coast” OR croatia OR cuba OR cyprus OR “czech republic” OR czechoslovakia OR djibouti OR “french somaliland” OR dominica OR “dominican republic” OR ecuador OR egypt OR “united arab republic” OR “el salvador” OR “equatorial guinea” OR “spanish guinea” OR eritrea OR estonia OR eswatini OR swaziland OR ethiopia OR fiji OR gabon OR “gabonese republic” OR gambia OR georgia OR georgian OR ghana OR “gold coast” OR gibraltar OR greece OR grenada OR guam OR guatemala OR guinea OR guyana OR guiana OR haiti OR hispaniola OR honduras OR hungary OR india OR indonesia OR timor OR iran OR iraq OR “isle of man” OR jamaica OR jordan OR kazakhstan OR kazakh OR kenya OR korea OR kosovo OR kyrgyzstan OR kirghizia OR kirgizstan OR “kyrgyz republic” OR kirghiz OR laos OR “lao pdr” OR “lao peoples democratic republic” OR latvia OR lebanon OR lesotho OR basutoland OR liberia OR libya OR “libyan arab jamahiriya” OR lithuania OR macau OR macao OR macedonia OR madagascar OR “malagasy republic” OR malawi OR nyasaland OR malaysia OR maldives OR “indian ocean” OR mali OR malta OR micronesia OR kiribati OR “marshall islands” OR nauru OR “northern mariana islands” OR palau OR tuvalu OR mauritania OR mauritius OR mexico OR moldova OR moldovian OR mongolia OR montenegro OR morocco OR ifni OR mozambique OR “portuguese east africa” OR myanmar OR burma OR namibia OR nepal OR “netherlands antilles” OR nicaragua OR niger OR nigeria OR oman OR muscat OR pakistan OR panama OR “papua new guinea” OR paraguay OR peru OR philippines OR philipines OR phillipines OR phillippines OR poland OR “polish peoples republic” OR portugal OR “portuguese republic” OR “puerto rico” OR romania OR russia OR “russian federation” OR ussr OR “soviet union” OR “union of soviet socialist republics” OR rwanda OR ruanda OR samoa OR “pacific islands” OR polynesia OR “samoan islands” OR “sao tome” AND principe OR “saudi arabia” OR senegal OR serbia OR seychelles OR “sierra leone” OR slovakia OR “slovak republic” OR slovenia OR melanesia OR “solomon island” OR “solomon islands” OR “norfolk island” OR somalia OR “south africa” OR “south sudan” OR “sri lanka” OR ceylon OR “saint kitts” AND nevis OR “st kitts” AND nevis OR “saint lucia” OR “st lucia” OR “saint vincent” OR “st vincent” OR grenadines OR sudan OR suriname OR surinam OR Syrian Arab Republic OR “syrian arab republic” OR tajikistan OR tadjikistan OR tadzhikistan OR tadzhik OR tanzania OR tanganyika OR thailand OR siam OR “timor leste” OR “east timor” OR togo OR “togolese republic” OR tonga OR trinidad OR tobago OR tunisia OR turkey OR turkmenistan OR turkmen OR uganda OR ukraine OR uruguay OR uzbekistan OR uzbek OR vanuatu OR “new hebrides” OR venezuela OR vietnam OR “viet nam” OR “middle east” OR “west bank” OR gaza OR palestine OR yemen OR yugoslavia OR zambia OR zimbabwe OR “northern rhodesia” OR “global south” OR “africa south of the sahara” OR “sub saharan africa” OR “subsaharan africa” OR “central africa” OR “north africa” OR “northern africa” OR magreb OR maghrib OR sahara OR “southern africa” OR “east africa” OR “eastern africa” OR “west africa” OR “western africa” OR “west indies” OR “indian ocean islands” OR caribbean OR “central america” OR “latin america” OR “south america” OR “central asia” OR “north asia” OR “northern asia” OR “southeastern asia” OR “south eastern asia” OR “southeast asia” OR “south east asia” OR “western asia” OR “east europe” OR “eastern europe” OR “developing country” OR “developing countries” OR “developing nation” OR “developing nations” OR “developing population” OR “developing populations” OR “developing world” OR “less developed country” OR “less developed countries” OR “less developed nation” OR “less developed nations” OR “less developed world” OR “lesser developed countries” OR “lesser developed nations” OR “under developed country” OR “under developed countries” OR “under developed nations” OR “under developed world” OR “underdeveloped country” OR “underdeveloped countries” OR “underdeveloped nation” OR “underdeveloped nations” OR “underdeveloped population” OR “underdeveloped populations” OR “underdeveloped world” OR “middle income country” OR “middle income countries” OR “middle income nation” OR “middle income nations” OR “middle income population” OR “middle income populations” OR “low income country” OR “low income countries” OR “low income nation” OR “low income nations” OR “low income population” OR “low income populations” OR “lower income country” OR “lower income countries” OR “lower income nations” OR “lower income population” OR “lower income populations” OR “underserved countries” OR “underserved nations” OR “underserved population” OR “underserved populations” OR “under served population” OR “under served populations” OR “deprived countries” OR “deprived population” OR “deprived populations” OR “poor country” OR “poor countries” OR “poor nation” OR “poor nations” OR “poor population” OR “poor populations” OR “poor world” OR “poorer countries” OR “poorer nations” OR “poorer population” OR “poorer populations” OR “developing economy” OR “developing economies” OR “less developed economy” OR “less developed economies” OR “underdeveloped economies” OR “middle income economy” OR “middle income economies” OR “low income economy” OR “low income economies” OR “lower income economies” OR “low gdp” OR “low gnp” OR “low gross domestic” OR “low gross national” OR “lower gdp” OR “lower gross domestic” OR lmic OR lmics OR “third world” OR “lami country” OR “lami countries” OR “transitional country” OR “transitional countries” OR “emerging economies” OR “emerging nation” OR “emerging nations”)

**APA PsycINFO®**

(Medical Overuse.ti,ab. OR Overmedicalization.ti,ab. OR Overmedicalisation.ti,ab. OR Overtreatment.ti,ab. OR Over-treatment.ti,ab. OR ((Overuse.ti,ab. OR Unnecessary.ti. OR Unwarranted.ti,ab. OR Inappropriate.ti. OR Deprescribing.ti,ab. OR De-implementation.ti,ab. OR Deimplementation.ti,ab.) AND (Medication.ti,ab. OR Therapeutic.ti,ab. OR Therapeutics.ti,ab. OR Antibiotics.ti,ab. OR Medicine.ti. OR Medicines.ti,ab. OR Prescriptions.ti,ab. OR “Pharmacological treatment.”ti,ab. OR “Pharmacological treatments.”ti,ab.)))

AND

(afghanistan.mp. OR albania.mp. OR algeria.mp. OR “american samoa.”mp. OR angola.mp. OR antigua.mp. OR barbuda.mp. OR argentina.mp. OR armenia.mp. OR armenian.mp. OR aruba.mp. OR azerbaijan.mp. OR bahrain.mp. OR bangladesh.mp. OR barbados.mp. OR belarus.mp. OR byelarus.mp. OR belorussia.mp. OR byelorussian.mp. OR belize.mp. OR “british honduras.”mp. OR benin.mp. OR dahomey.mp. OR bhutan.mp. OR bolivia.mp. OR bosnia.mp. OR herzegovina.mp. OR botswana.mp. OR bechuanaland.mp. OR brazil.mp. OR brasil.mp. OR bulgaria.mp. OR “burkina faso.”mp. OR “burkina fasso.”mp. OR “upper volta.”mp. OR burundi.mp. OR urundi.mp. OR “cabo verde.”mp. OR “cape verde.”mp. OR cambodia.mp. OR kampuchea.mp. OR “khmer republic.”mp. OR cameroon.mp. OR cameron.mp. OR cameroun.mp. OR “central african republic.”mp. OR “ubangi shari.”mp. OR chad.mp. OR chile.mp. OR china.mp. OR colombia.mp. OR comoros.mp. OR “comoro islands.”mp. OR mayotte.mp. OR congo.mp. OR zaire.mp. OR “costa rica.”mp. OR “cote d’ivoire.”mp. OR “cote d’ ivoire.”mp. OR “cote divoire.”mp. OR “cote d ivoire.”mp. OR “ivory coast.”mp. OR croatia.mp. OR cuba.mp. OR cyprus.mp. OR “czech republic.”mp. OR czechoslovakia.mp. OR djibouti.mp. OR “french somaliland.”mp. OR dominica.mp. OR “dominican republic.”mp. OR ecuador.mp. OR egypt.mp. OR “united arab republic.”mp. OR “el salvador.”mp. OR “equatorial guinea.”mp. OR “spanish guinea.”mp. OR eritrea.mp. OR estonia.mp. OR eswatini.mp. OR swaziland.mp. OR ethiopia.mp. OR fiji.mp. OR gabon.mp. OR “gabonese republic.”mp. OR gambia.mp. OR georgia.mp. OR georgian.mp. OR ghana.mp. OR “gold coast.”mp. OR gibraltar.mp. OR greece.mp. OR grenada.mp. OR guam.mp. OR guatemala.mp. OR guinea.mp. OR guyana.mp. OR guiana.mp. OR haiti.mp. OR hispaniola.mp. OR honduras.mp. OR hungary.mp. OR india.mp. OR indonesia.mp. OR timor.mp. OR iran.mp. OR iraq.mp. OR “isle of man.”mp. OR jamaica.mp. OR jordan.mp. OR kazakhstan.mp. OR kazakh.mp. OR kenya.mp. OR korea.mp. OR kosovo.mp. OR kyrgyzstan.mp. OR kirghizia.mp. OR kirgizstan.mp. OR “kyrgyz republic.”mp. OR kirghiz.mp. OR laos.mp. OR “lao pdr.”mp. OR “lao people's democratic republic.”mp. OR latvia.mp. OR lebanon.mp. OR lesotho.mp. OR basutoland.mp. OR liberia.mp. OR libya.mp. OR “libyan arab jamahiriya.”mp. OR lithuania.mp. OR macau.mp. OR macao.mp. OR macedonia.mp. OR madagascar.mp. OR “malagasy republic.”mp. OR malawi.mp. OR nyasaland.mp. OR malaysia.mp. OR maldives.mp. OR “indian ocean.”mp. OR mali.mp. OR malta.mp. OR micronesia.mp. OR kiribati.mp. OR “marshall islands.”mp. OR nauru.mp. OR “northern mariana islands.”mp. OR palau.mp. OR tuvalu.mp. OR mauritania.mp. OR mauritius.mp. OR mexico.mp. OR moldova.mp. OR moldovian.mp. OR mongolia.mp. OR montenegro.mp. OR morocco.mp. OR ifni.mp. OR mozambique.mp. OR “portuguese east africa.”mp. OR myanmar.mp. OR burma.mp. OR namibia.mp. OR nepal.mp. OR “netherlands antilles.”mp. OR nicaragua.mp. OR niger.mp. OR nigeria.mp. OR oman.mp. OR muscat.mp. OR pakistan.mp. OR panama.mp. OR “papua new guinea.”mp. OR paraguay.mp. OR peru.mp. OR philippines.mp. OR philipines.mp. OR phillipines.mp. OR phillippines.mp. OR poland.mp. OR “polish people's republic.”mp. OR portugal.mp. OR “portuguese republic.”mp. OR “puerto rico.”mp. OR romania.mp. OR russia.mp. OR “russian federation.”mp. OR ussr.mp. OR “soviet union.”mp. OR “union of soviet socialist republics.”mp. OR rwanda.mp. OR ruanda.mp. OR samoa.mp. OR “pacific islands.”mp. OR polynesia.mp. OR “samoan islands.”mp. OR “sao tome” AND principe.mp. OR “saudi arabia.”mp. OR senegal.mp. OR serbia.mp. OR seychelles.mp. OR “sierra leone.”mp. OR slovakia.mp. OR “slovak republic.”mp. OR slovenia.mp. OR melanesia.mp. OR “solomon island.”mp. OR “solomon islands.”mp. OR “norfolk island.”mp. OR somalia.mp. OR “south africa.”mp. OR “south sudan.”mp. OR “sri lanka.”mp. OR ceylon.mp. OR “saint kitts” AND nevis.mp. OR “st kitts” AND nevis.mp. OR “saint lucia.”mp. OR “st lucia.”mp. OR “saint vincent.”mp. OR “st vincent.”mp. OR grenadines.mp. OR sudan.mp. OR suriname.mp. OR surinam.mp. OR Syrian Arab Republic.mp. OR “syrian arab republic.”mp. OR tajikistan.mp. OR tadjikistan.mp. OR tadzhikistan.mp. OR tadzhik.mp. OR tanzania.mp. OR tanganyika.mp. OR thailand.mp. OR siam.mp. OR “timor leste.”mp. OR “east timor.”mp. OR togo.mp. OR “togolese republic.”mp. OR tonga.mp. OR trinidad.mp. OR tobago.mp. OR tunisia.mp. OR turkey.mp. OR turkmenistan.mp. OR turkmen.mp. OR uganda.mp. OR ukraine.mp. OR uruguay.mp. OR uzbekistan.mp. OR uzbek.mp. OR vanuatu.mp. OR “new hebrides.”mp. OR venezuela.mp. OR vietnam.mp. OR “viet nam.”mp. OR “middle east.”mp. OR “west bank.”mp. OR gaza.mp. OR palestine.mp. OR yemen.mp. OR yugoslavia.mp. OR zambia.mp. OR zimbabwe.mp. OR “northern rhodesia.”mp. OR “global south.”mp. OR “africa south of the sahara.”mp. OR “sub saharan africa.”mp. OR “subsaharan africa.”mp. OR “central africa.”mp. OR “north africa.”mp. OR “northern africa.”mp. OR magreb.mp. OR maghrib.mp. OR sahara.mp. OR “southern africa.”mp. OR “east africa.”mp. OR “eastern africa.”mp. OR “west africa.”mp. OR “western africa.”mp. OR “west indies.”mp. OR “indian ocean islands.”mp. OR caribbean.mp. OR “central america.”mp. OR “latin america.”mp. OR “south america.”mp. OR “central asia.”mp. OR “north asia.”mp. OR “northern asia.”mp. OR “southeastern asia.”mp. OR “south eastern asia.”mp. OR “southeast asia.”mp. OR “south east asia.”mp. OR “western asia.”mp. OR “east europe.”mp. OR “eastern europe.”mp. OR “developing country.”mp. OR “developing countries.”mp. OR “developing nation.”mp. OR “developing nations.”mp. OR “developing population.”mp. OR “developing populations.”mp. OR “developing world.”mp. OR “less developed country.”mp. OR “less developed countries.”mp. OR “less developed nation.”mp. OR “less developed nations.”mp. OR “less developed world.”mp. OR “lesser developed countries.”mp. OR “lesser developed nations.”mp. OR “under developed country.”mp. OR “under developed countries.”mp. OR “under developed nations.”mp. OR “under developed world.”mp. OR “underdeveloped country.”mp. OR “underdeveloped countries.”mp. OR “underdeveloped nation.”mp. OR “underdeveloped nations.”mp. OR “underdeveloped population.”mp. OR “underdeveloped populations.”mp. OR “underdeveloped world.”mp. OR “middle income country.”mp. OR “middle income countries.”mp. OR “middle income nation.”mp. OR “middle income nations.”mp. OR “middle income population.”mp. OR “middle income populations.”mp. OR “low income country.”mp. OR “low income countries.”mp. OR “low income nation.”mp. OR “low income nations.”mp. OR “low income population.”mp. OR “low income populations.”mp. OR “lower income country.”mp. OR “lower income countries.”mp. OR “lower income nations.”mp. OR “lower income population.”mp. OR “lower income populations.”mp. OR “underserved countries.”mp. OR “underserved nations.”mp. OR “underserved population.”mp. OR “underserved populations.”mp. OR “under served population.”mp. OR “under served populations.”mp. OR “deprived countries.”mp. OR “deprived population.”mp. OR “deprived populations.”mp. OR “poor country.”mp. OR “poor countries.”mp. OR “poor nation.”mp. OR “poor nations.”mp. OR “poor population.”mp. OR “poor populations.”mp. OR “poor world.”mp. OR “poorer countries.”mp. OR “poorer nations.”mp. OR “poorer population.”mp. OR “poorer populations.”mp. OR “developing economy.”mp. OR “developing economies.”mp. OR “less developed economy.”mp. OR “less developed economies.”mp. OR “underdeveloped economies.”mp. OR “middle income economy.”mp. OR “middle income economies.”mp. OR “low income economy.”mp. OR “low income economies.”mp. OR “lower income economies.”mp. OR “low gdp.”mp. OR “low gnp.”mp. OR “low gross domestic.”mp. OR “low gross national.”mp. OR “lower gdp.”mp. OR “lower gross domestic.”mp. OR lmic.mp. OR lmics.mp. OR “third world.”mp. OR “lami country.”mp. OR “lami countries.”mp. OR “transitional country.”mp. OR “transitional countries.”mp. OR “emerging economies.”mp. OR “emerging nation.”mp. OR “emerging nations.”mp.)

**Global Index Medicus (WPRIM (Western Pacific); LILACS (Americas); IMSEAR (South-East Asia); IMEMR (Eastern Mediterranean); AIM (Africa))**

(“Medical Overuse” OR Overmedicalization OR Overmedicalisation OR (ti:(Overtreatment OR Over-treatment OR Overuse OR Deprescribing OR De-implementation OR Deimplementation)) AND (tw:(Medication OR Therapeutic OR Therapeutics OR Antibiotics OR Medicine OR Medicines OR Prescriptions OR “Pharmacological treatment” OR “Pharmacological treatments”)))

### Eligibility criteria

We included studies from one or more low- and middle-income countries^13^ with a major focus on any of the four medication overuse themes: (i) extent of overuse; (ii) drivers and factors related to overuse; (iii) consequences of overuse; and (iv) solutions addressing the overuses. We included studies reporting data on low- and middle-income countries and high-income countries if data pertaining to low- and middle-income countries could be analysed separately, or the majority (≥ 75%) of reported data were from low- and middle-income countries. For this review, we used a broad commonly used definition of overuse of medications as the provision of medications which are unlikely to benefit the patient given the harms, cost, available alternatives or preferences of the patient – including unnecessary, inappropriate and potentially inappropriate medications.^1^^,^^2^ We also accepted operational definitions and assessments of primary study authors to estimate the extent of overuse of medications – including using Beers criteria® for Potentially Inappropriate Medication Use in Older Adults, or the screening tool of older persons’ prescriptions (STOPP), or appropriateness as judged by local guidelines.

We used the World Bank categorization from 2021^13^ to define low- and middle-income countries (available in data repository).^14^

We included quantitative interventional and observational studies as well as qualitative studies, both primary and secondary studies, such as systematic reviews of eligible primary studies, and peer-reviewed articles or grey literature (e.g. eligible reports from governmental and nongovernmental organizations and conference abstracts). We included studies regardless of clinical setting (inpatient or outpatient, or the level of care), type of medications assessed (e.g. whether prescribed or nonprescription medications) and population type. We excluded case reports and case series, non-research opinion or analysis, literature reviews, conference abstracts with limited information to judge eligibility or to use for evidence synthesis, and studies where overuse of medications was not a major or primary focus or finding of the study.

### Study selection

A total of nine pairs of reviewers independently screened titles and abstracts, and subsequently full text, using an open-access web-based tool.^9^ Any disagreements were resolved, at all stages, by discussion or reference to a third author.

### Data extraction

We used a prospectively developed and piloted data extraction form. A single reviewer extracted data on (i) study characteristics including sample size and study design; (ii) overuse of medications including conditions and medication studied; (iii) main study themes (extent, drivers, consequences and solutions); and (iv) relevant key findings. For secondary studies (e.g. systematic reviews), we extracted data from summarized information of included studies and not directly from primary studies.

### Data synthesis

We grouped studies by a priori defined groups: (i) the major focus or the four main themes; (ii) condition (i.e. per Global Burden of Disease classification); (iii) countries or country income level; and (iv) medication classes (i.e. by the major categories in the United States Pharmacopeial Medicare Model Guidelines v6.0).

## Results

**Fig. 1:**
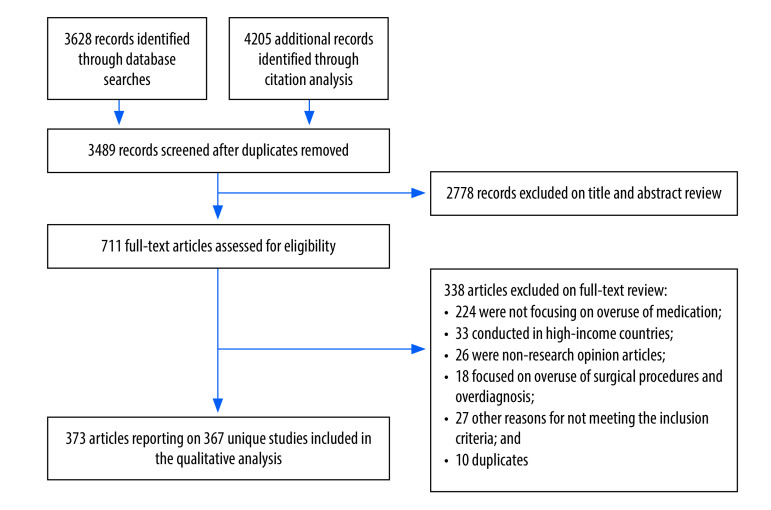
Flowchart of the selection of articles included in study on overuse of medications in low- and middle-income countries

We identified a total of 3489 unique records. After screening titles and abstracts, we identified 711 records for full-text screening. Of the full-text articles screened, 338 were excluded with reasons recorded, and we included 373 articles^15^^–^^387^ reporting on a total of 367 unique studies (Fig. 1).^15^^–^^381^

### Characteristics of studies

**Fig. 2:**
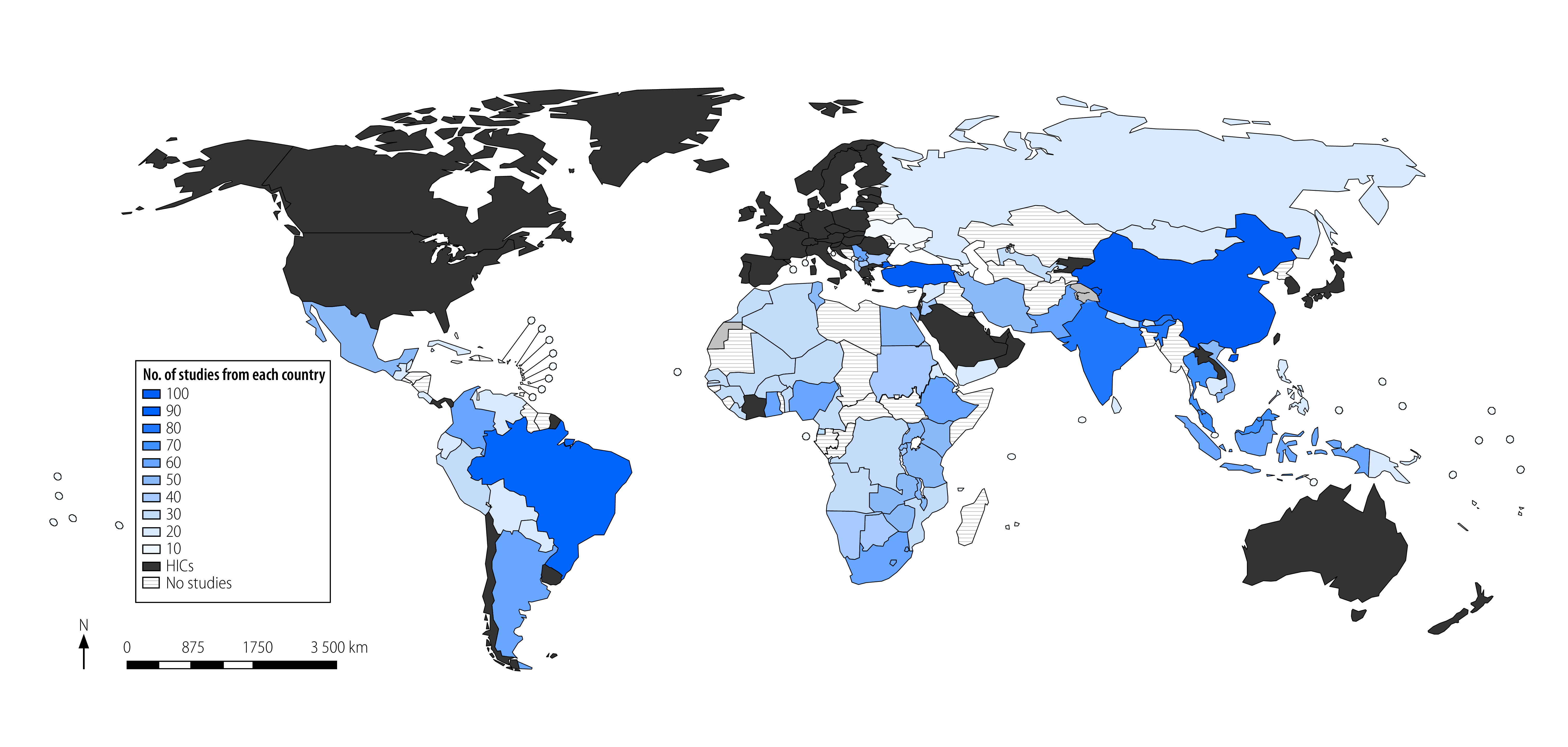
Studies of overuse of medications in low- and middle-income countries

The 367 included studies collectively reported on 5 120 468 prescribed or nonprescription medications (median: 1185; interquartile range, IQR: 333–3017) and more than 5 322 693 participants (median: 495; IQR: 222–1522) from 80 low- and middle-income countries. Of all 134 low- and middle-income countries, we found studies from 17 (58.6%) of all 29 low-income countries, 31 (62.0%) of all 50 lower-middle-income countries, and 33 (60.0%) of all 55 upper-middle-income countries. Twenty-one studies were multinational. Of the 346 studies originating from single countries, 232 (67.1%) were from upper-middle income countries and 99 (28.6%) from the East Asia and Pacific region (Table 1; Fig. 2).

**Table 1:** Characteristics of the 367 included studies in the scoping review on overuse of medications in low- and middle-income countries

Study characteristic	Study reference	No. (%) of studies
**Publication year**		
1990–2000	^23^ ^,^ ^50^ ^,^ ^67^ ^,^ ^113^ ^,^ ^168^ ^,^ ^185^ ^,^ ^187^ ^,^ ^201^ ^,^ ^233^ ^,^ ^248^	10 (2.7)
2001–2010	^18^ ^,^ ^22^ ^,^ ^49^ ^,^ ^54^ ^,^ ^56^ ^,^ ^57^ ^,^ ^71^ ^,^ ^85^ ^,^ ^95^ ^,^ ^110^ ^,^ ^145^ ^,^ ^159^ ^,^ ^169^ ^,^ ^172^ ^,^ ^181^ ^,^ ^193^ ^,^ ^195^ ^,^ ^202^ ^,^ ^213^ ^,^ ^256^ ^,^ ^264^ ^,^ ^270^ ^,^ ^285^ ^,^ ^289^ ^,^ ^291^ ^,^ ^295^ ^,^ ^318^	27 (7.4)
2011–2021	^15^ ^–^ ^17^ ^,^ ^19^ ^–^ ^21^ ^,^ ^24^ ^–^ ^48^ ^,^ ^51^ ^–^ ^53^ ^,^ ^55^ ^,^ ^58^ ^–^ ^66^ ^,^ ^68^ ^–^ ^70^ ^,^ ^72^ ^–^ ^84^ ^,^ ^86^ ^–^ ^94^ ^,^ ^96^ ^–^ ^109^ ^,^ ^111^ ^,^ ^112^ ^,^ ^114^ ^–^ ^144^ ^,^ ^146^ ^–^ ^158^ ^,^ ^160^ ^–^ ^167^ ^,^ ^170^ ^,^ ^171^ ^,^ ^173^ ^–^ ^180^ ^,^ ^182^ ^–^ ^184^ ^,^ ^186^ ^,^ ^188^ ^–^ ^192^ ^,^ ^194^ ^,^ ^196^ ^–^ ^200^ ^,^ ^203^ ^–^ ^212^ ^,^ ^214^ ^–^ ^232^ ^,^ ^234^ ^–^ ^247^ ^,^ ^249^ ^–^ ^255^ ^,^ ^257^ ^–^ ^263^ ^,^ ^265^ ^–^ ^269^ ^,^ ^271^ ^–^ ^284^ ^,^ ^286^ ^–^ ^288^ ^,^ ^290^ ^,^ ^292^ ^–^ ^294^ ^,^ ^296^ ^–^ ^317^ ^,^ ^319^ ^–^ ^381^	330 (89.9)
**Language of publication**		
English	^15^ ^,^ ^17^ ^–^ ^21^ ^,^ ^24^ ^–^ ^49^ ^,^ ^51^ ^–^ ^66^ ^,^ ^68^ ^–^ ^91^ ^,^ ^93^ ^–^ ^132^ ^,^ ^134^ ^–^ ^165^ ^,^ ^167^ ^,^ ^168^ ^,^ ^170^ ^–^ ^213^ ^,^ ^215^ ^–^ ^224^ ^,^ ^226^ ^,^ ^228^ ^–^ ^235^ ^,^ ^238^ ^–^ ^266^ ^,^ ^268^ ^–^ ^270^ ^,^ ^272^ ^,^ ^273^ ^,^ ^275^ ^–^ ^289^ ^,^ ^291^ ^–^ ^329^ ^,^ ^331^ ^–^ ^381^	347 (94.6)
Spanish	^50^ ^,^ ^90^ ^,^ ^133^ ^,^ ^169^ ^,^ ^227^ ^,^ ^236^ ^,^ ^237^ ^,^ ^267^ ^,^ ^271^ ^,^ ^274^ ^,^ ^290^	11 (3)
French	^16^ ^,^ ^23^ ^,^ ^67^ ^,^ ^166^	4 (1.1)
Portuguese	^22^ ^,^ ^92^ ^,^ ^214^ ^,^ ^225^	4 (1.1)
Russian	^330^	1 (0.3)
**No. of countries included in the study **		
One	^15^ ^–^ ^23^ ^,^ ^25^ ^–^ ^52^ ^,^ ^54^ ^–^ ^57^ ^,^ ^59^ ^–^ ^72^ ^,^ ^74^ ^–^ ^82^ ^,^ ^84^ ^,^ ^85^ ^,^ ^87^ ^–^ ^138^ ^,^ ^141^ ^–^ ^147^ ^,^ ^149^ ^–^ ^169^ ^,^ ^171^ ^–^ ^179^ ^,^ ^181^ ^–^ ^200^ ^,^ ^202^ ^–^ ^208^ ^,^ ^211^ ^–^ ^221^ ^,^ ^223^ ^–^ ^331^ ^,^ ^334^ ^–^ ^346^ ^,^ ^348^ ^–^ ^381^	346 (94.3)
Multiple countries	^24^ ^,^ ^53^ ^,^ ^58^ ^,^ ^73^ ^,^ ^83^ ^,^ ^86^ ^,^ ^139^ ^,^ ^140^ ^,^ ^148^ ^,^ ^170^ ^,^ ^180^ ^,^ ^201^ ^,^ ^209^ ^,^ ^210^ ^,^ ^222^ ^,^ ^242^ ^,^ ^297^ ^,^ ^326^ ^,^ ^332^ ^,^ ^333^ ^,^ ^347^	21 (5.7)
**Country income level **		
Low income	^18^ ^,^ ^20^ ^,^ ^21^ ^,^ ^38^ ^,^ ^67^ ^,^ ^75^ ^,^ ^77^ ^,^ ^111^ ^,^ ^127^ ^,^ ^129^ ^,^ ^144^ ^,^ ^146^ ^,^ ^166^ ^,^ ^183^ ^,^ ^230^ ^,^ ^231^ ^,^ ^245^ ^,^ ^249^ ^,^ ^256^ ^,^ ^262^ ^,^ ^316^ ^,^ ^329^ ^,^ ^335^ ^,^ ^337^ ^,^ ^343^	25 (6.8)
Lower-middle income	^16^ ^,^ ^17^ ^,^ ^23^ ^,^ ^25^ ^,^ ^27^ ^–^ ^29^ ^,^ ^31^ ^,^ ^43^ ^,^ ^45^ ^,^ ^51^ ^,^ ^52^ ^,^ ^55^ ^,^ ^61^ ^,^ ^64^ ^,^ ^68^ ^,^ ^69^ ^,^ ^71^ ^,^ ^72^ ^,^ ^74^ ^,^ ^78^ ^,^ ^79^ ^,^ ^81^ ^,^ ^85^ ^,^ ^93^ ^,^ ^99^ ^,^ ^108^ ^,^ ^114^ ^,^ ^117^ ^,^ ^122^ ^,^ ^124^ ^,^ ^130^ ^,^ ^131^ ^,^ ^134^ ^,^ ^145^ ^,^ ^150^ ^,^ ^155^ ^,^ ^159^ ^,^ ^167^ ^,^ ^168^ ^,^ ^175^ ^,^ ^179^ ^,^ ^181^ ^,^ ^182^ ^,^ ^185^ ^,^ ^186^ ^,^ ^191^ ^–^ ^194^ ^,^ ^197^ ^,^ ^220^ ^,^ ^226^ ^,^ ^229^ ^,^ ^234^ ^,^ ^235^ ^,^ ^240^ ^,^ ^241^ ^,^ ^246^ ^,^ ^247^ ^,^ ^251^ ^–^ ^253^ ^,^ ^260^ ^,^ ^261^ ^,^ ^263^ ^,^ ^265^ ^,^ ^275^ ^,^ ^281^ ^,^ ^284^ ^,^ ^287^ ^,^ ^288^ ^,^ ^293^ ^,^ ^295^ ^,^ ^298^ ^,^ ^301^ ^,^ ^302^ ^,^ ^307^ ^,^ ^308^ ^,^ ^310^ ^–^ ^313^ ^,^ ^315^ ^,^ ^319^ ^,^ ^339^ ^,^ ^346^ ^,^ ^353^ ^,^ ^356^	89 (24.3)
Upper-middle income	^15^ ^,^ ^19^ ^,^ ^22^ ^,^ ^26^ ^,^ ^30^ ^,^ ^32^ ^–^ ^37^ ^,^ ^39^ ^–^ ^42^ ^,^ ^44^ ^,^ ^46^ ^,^ ^47^ ^,^ ^49^ ^–^ ^51^ ^,^ ^54^ ^,^ ^56^ ^,^ ^57^ ^,^ ^59^ ^,^ ^60^ ^,^ ^62^ ^,^ ^63^ ^,^ ^65^ ^,^ ^66^ ^,^ ^70^ ^,^ ^76^ ^,^ ^80^ ^,^ ^82^ ^,^ ^84^ ^,^ ^87^ ^–^ ^92^ ^,^ ^94^ ^–^ ^98^ ^,^ ^100^ ^–^ ^107^ ^,^ ^109^ ^,^ ^110^ ^,^ ^112^ ^,^ ^113^ ^,^ ^115^ ^,^ ^116^ ^,^ ^118^ ^–^ ^121^ ^,^ ^123^ ^,^ ^125^ ^,^ ^126^ ^,^ ^128^ ^,^ ^132^ ^,^ ^133^ ^,^ ^135^ ^–^ ^138^ ^,^ ^141^ ^–^ ^143^ ^,^ ^147^ ^,^ ^149^ ^,^ ^151^ ^–^ ^154^ ^,^ ^156^ ^–^ ^158^ ^,^ ^160^ ^–^ ^165^ ^,^ ^169^ ^,^ ^171^ ^–^ ^174^ ^,^ ^176^ ^–^ ^178^ ^,^ ^184^ ^,^ ^187^ ^–^ ^190^ ^,^ ^195^ ^,^ ^196^ ^,^ ^198^ ^–^ ^200^ ^,^ ^202^ ^–^ ^208^ ^,^ ^211^ ^–^ ^219^ ^,^ ^221^ ^,^ ^223^ ^–^ ^225^ ^,^ ^227^ ^,^ ^228^ ^,^ ^232^ ^,^ ^233^ ^,^ ^236^ ^–^ ^239^ ^,^ ^243^ ^,^ ^244^ ^,^ ^248^ ^,^ ^250^ ^,^ ^254^ ^,^ ^255^ ^,^ ^257^ ^–^ ^259^ ^,^ ^264^ ^,^ ^266^ ^–^ ^274^ ^,^ ^276^ ^–^ ^280^ ^,^ ^282^ ^–^ ^286^ ^,^ ^289^ ^–^ ^292^ ^,^ ^294^ ^,^ ^296^ ^,^ ^299^ ^,^ ^300^ ^,^ ^303^ ^–^ ^306^ ^,^ ^309^ ^,^ ^314^ ^,^ ^317^ ^,^ ^318^ ^,^ ^320^ ^–^ ^325^ ^,^ ^327^ ^,^ ^328^ ^,^ ^330^ ^,^ ^331^ ^,^ ^334^ ^,^ ^336^ ^,^ ^338^ ^,^ ^340^ ^–^ ^342^ ^,^ ^344^ ^,^ ^345^ ^,^ ^348^ ^–^ ^352^ ^,^ ^354^ ^,^ ^355^ ^,^ ^357^ ^–^ ^381^	232 (63.2)
**World Bank geographical region^a^**		
Sub-Saharan Africa	^20^ ^,^ ^21^ ^,^ ^25^ ^,^ ^27^ ^,^ ^28^ ^,^ ^31^ ^,^ ^45^ ^,^ ^55^ ^,^ ^67^ ^,^ ^75^ ^–^ ^77^ ^,^ ^108^ ^,^ ^111^ ^,^ ^122^ ^,^ ^124^ ^,^ ^127^ ^,^ ^129^ ^–^ ^131^ ^,^ ^135^ ^,^ ^144^ ^,^ ^146^ ^,^ ^166^ ^,^ ^168^ ^,^ ^183^ ^,^ ^185^ ^,^ ^197^ ^,^ ^220^ ^,^ ^226^ ^,^ ^228^ ^,^ ^230^ ^,^ ^231^ ^,^ ^245^ ^,^ ^249^ ^,^ ^256^ ^,^ ^261^ ^–^ ^263^ ^,^ ^282^ ^,^ ^287^ ^,^ ^288^ ^,^ ^295^ ^,^ ^316^ ^,^ ^329^ ^,^ ^335^ ^,^ ^337^ ^,^ ^343^ ^,^ ^349^	49 (13.4)
East Asia and Pacific	^15^ ^,^ ^19^ ^,^ ^26^ ^,^ ^30^ ^,^ ^33^ ^,^ ^34^ ^,^ ^50^ ^,^ ^56^ ^,^ ^65^ ^,^ ^66^ ^,^ ^97^ ^,^ ^98^ ^,^ ^100^ ^–^ ^103^ ^,^ ^106^ ^,^ ^115^ ^,^ ^117^ ^–^ ^119^ ^,^ ^121^ ^,^ ^132^ ^,^ ^149^ ^,^ ^151^ ^,^ ^154^ ^–^ ^156^ ^,^ ^160^ ^–^ ^162^ ^,^ ^164^ ^,^ ^165^ ^,^ ^167^ ^,^ ^172^ ^,^ ^174^ ^,^ ^177^ ^,^ ^179^ ^,^ ^190^ ^,^ ^196^ ^,^ ^199^ ^,^ ^200^ ^,^ ^203^ ^–^ ^208^ ^,^ ^211^ ^,^ ^212^ ^,^ ^215^ ^,^ ^217^ ^,^ ^218^ ^,^ ^223^ ^,^ ^233^ ^,^ ^239^ ^,^ ^252^ ^,^ ^253^ ^,^ ^255^ ^,^ ^257^ ^,^ ^260^ ^,^ ^264^ ^,^ ^268^ ^,^ ^276^ ^,^ ^277^ ^,^ ^279^ ^,^ ^285^ ^,^ ^307^ ^,^ ^309^ ^,^ ^318^ ^,^ ^323^ ^,^ ^327^ ^,^ ^328^ ^,^ ^331^ ^,^ ^338^ ^,^ ^351^ ^,^ ^352^ ^,^ ^356^ ^–^ ^369^ ^,^ ^371^ ^,^ ^372^ ^,^ ^376^ ^–^ ^381^	99 (27.0)
Europe and Central Asia	^32^ ^,^ ^35^ ^,^ ^39^ ^,^ ^51^ ^,^ ^54^ ^,^ ^59^ ^,^ ^60^ ^,^ ^62^ ^,^ ^70^ ^,^ ^80^ ^,^ ^84^ ^,^ ^91^ ^,^ ^94^ ^,^ ^95^ ^,^ ^107^ ^,^ ^125^ ^,^ ^126^ ^,^ ^128^ ^,^ ^150^ ^,^ ^152^ ^,^ ^157^ ^,^ ^158^ ^,^ ^176^ ^,^ ^178^ ^,^ ^187^ ^–^ ^189^ ^,^ ^198^ ^,^ ^202^ ^,^ ^250^ ^,^ ^265^ ^,^ ^266^ ^,^ ^276^ ^,^ ^283^ ^,^ ^296^ ^,^ ^303^ ^,^ ^304^ ^,^ ^306^ ^,^ ^310^ ^,^ ^317^ ^,^ ^320^ ^,^ ^322^ ^,^ ^324^ ^,^ ^325^ ^,^ ^330^ ^,^ ^334^ ^,^ ^336^ ^,^ ^340^ ^,^ ^342^ ^,^ ^344^ ^,^ ^345^ ^,^ ^350^ ^,^ ^370^ ^,^ ^373^	54 (14.7)
Latin America and Caribbean	^22^ ^,^ ^37^ ^,^ ^40^ ^,^ ^42^ ^,^ ^44^ ^,^ ^63^ ^,^ ^82^ ^,^ ^87^ ^,^ ^88^ ^,^ ^90^ ^,^ ^92^ ^,^ ^104^ ^,^ ^105^ ^,^ ^109^ ^,^ ^110^ ^,^ ^112^ ^,^ ^113^ ^,^ ^116^ ^,^ ^120^ ^,^ ^133^ ^,^ ^136^ ^–^ ^138^ ^,^ ^141^ ^–^ ^143^ ^,^ ^147^ ^,^ ^169^ ^,^ ^171^ ^,^ ^184^ ^,^ ^195^ ^,^ ^213^ ^,^ ^214^ ^,^ ^216^ ^,^ ^219^ ^,^ ^221^ ^,^ ^224^ ^,^ ^225^ ^,^ ^227^ ^,^ ^236^ ^–^ ^238^ ^,^ ^243^ ^,^ ^248^ ^,^ ^254^ ^,^ ^258^ ^,^ ^259^ ^,^ ^267^ ^,^ ^269^ ^,^ ^270^ ^,^ ^273^ ^,^ ^274^ ^,^ ^286^ ^,^ ^289^ ^,^ ^290^ ^,^ ^299^ ^,^ ^300^ ^,^ ^305^ ^,^ ^341^ ^,^ ^348^ ^,^ ^354^ ^,^ ^355^	62 (16.9)
Middle East and North Africa	^16^ ^–^ ^18^ ^,^ ^23^ ^,^ ^36^ ^,^ ^38^ ^,^ ^41^ ^,^ ^43^ ^,^ ^46^ ^,^ ^47^ ^,^ ^49^ ^,^ ^57^ ^,^ ^74^ ^,^ ^89^ ^,^ ^96^ ^,^ ^123^ ^,^ ^153^ ^,^ ^163^ ^,^ ^173^ ^,^ ^175^ ^,^ ^186^ ^,^ ^232^ ^,^ ^240^ ^,^ ^244^ ^,^ ^272^ ^,^ ^280^ ^,^ ^291^ ^–^ ^294^ ^,^ ^314^ ^,^ ^374^ ^,^ ^375^	33 (9.0)
South Asia	^29^ ^,^ ^48^ ^,^ ^52^ ^,^ ^61^ ^,^ ^64^ ^,^ ^68^ ^,^ ^69^ ^,^ ^71^ ^,^ ^72^ ^,^ ^78^ ^,^ ^79^ ^,^ ^81^ ^,^ ^85^ ^,^ ^93^ ^,^ ^99^ ^,^ ^114^ ^,^ ^134^ ^,^ ^145^ ^,^ ^159^ ^,^ ^181^ ^,^ ^182^ ^,^ ^191^ ^–^ ^194^ ^,^ ^229^ ^,^ ^234^ ^,^ ^235^ ^,^ ^241^ ^,^ ^246^ ^,^ ^247^ ^,^ ^251^ ^,^ ^271^ ^,^ ^275^ ^,^ ^281^ ^,^ ^284^ ^,^ ^298^ ^,^ ^301^ ^,^ ^302^ ^,^ ^308^ ^,^ ^310^ ^–^ ^313^ ^,^ ^315^ ^,^ ^319^ ^,^ ^339^ ^,^ ^346^ ^,^ ^353^	49 (13.4)
**Study design**		
Interventional (e.g. RCT)	^37^ ^,^ ^45^ ^,^ ^50^ ^,^ ^52^ ^,^ ^55^ ^,^ ^65^ ^,^ ^66^ ^,^ ^98^ ^,^ ^106^ ^,^ ^108^ ^,^ ^114^ ^,^ ^117^ ^,^ ^176^ ^,^ ^186^ ^,^ ^189^ ^,^ ^192^ ^,^ ^201^ ^,^ ^215^ ^,^ ^231^ ^,^ ^234^ ^,^ ^236^ ^,^ ^240^ ^,^ ^249^ ^,^ ^260^ ^,^ ^267^ ^,^ ^299^ ^,^ ^313^ ^,^ ^329^ ^,^ ^341^ ^,^ ^347^ ^,^ ^356^ ^,^ ^359^ ^,^ ^363^ ^,^ ^368^ ^,^ ^369^ ^,^ ^378^	36 (9.8)
Randomized trials (e.g. cluster RCTs)	^45^ ^,^ ^55^ ^,^ ^98^ ^,^ ^117^ ^,^ ^231^ ^,^ ^240^ ^,^ ^249^ ^,^ ^341^ ^,^ ^347^ ^,^ ^359^ ^,^ ^369^	11 (3.0)
Controlled studies	^37^ ^,^ ^52^ ^,^ ^66^ ^,^ ^106^ ^,^ ^201^ ^,^ ^267^ ^,^ ^299^ ^,^ ^313^ ^,^ ^368^ ^,^ ^378^	10 (2.7)
Before-and-after studies	^50^ ^,^ ^65^ ^,^ ^108^ ^,^ ^114^ ^,^ ^176^ ^,^ ^186^ ^,^ ^189^ ^,^ ^192^ ^,^ ^215^ ^,^ ^234^ ^,^ ^236^ ^,^ ^260^ ^,^ ^329^ ^,^ ^356^ ^,^ ^363^	15 (4.1)
Observational	^15^ ^–^ ^23^ ^,^ ^25^ ^–^ ^36^ ^,^ ^38^ ^,^ ^40^ ^,^ ^42^ ^–^ ^44^ ^,^ ^46^ ^,^ ^48^ ^,^ ^51^ ^,^ ^54^ ^,^ ^56^ ^–^ ^64^ ^,^ ^67^ ^–^ ^72^ ^,^ ^74^ ^–^ ^77^ ^,^ ^79^ ^–^ ^85^ ^,^ ^87^ ^–^ ^97^ ^,^ ^99^ ^–^ ^105^ ^,^ ^107^ ^,^ ^109^ ^–^ ^113^ ^,^ ^115^ ^,^ ^116^ ^,^ ^118^ ^–^ ^120^ ^,^ ^122^ ^–^ ^138^ ^,^ ^140^ ^–^ ^147^ ^,^ ^149^ ^–^ ^175^ ^,^ ^177^ ^–^ ^185^ ^,^ ^187^ ^,^ ^188^ ^,^ ^190^ ^,^ ^191^ ^,^ ^193^ ^–^ ^200^ ^,^ ^202^ ^–^ ^208^ ^,^ ^211^ ^–^ ^214^ ^,^ ^216^ ^–^ ^221^ ^,^ ^223^ ^–^ ^230^ ^,^ ^232^ ^,^ ^233^ ^,^ ^235^ ^,^ ^237^ ^–^ ^239^ ^,^ ^241^ ^–^ ^248^ ^,^ ^250^ ^–^ ^259^ ^,^ ^261^ ^–^ ^264^ ^,^ ^266^ ^,^ ^268^ ^–^ ^271^ ^,^ ^273^ ^–^ ^298^ ^,^ ^300^ ^–^ ^312^ ^,^ ^314^ ^–^ ^325^ ^,^ ^327^ ^,^ ^328^ ^,^ ^330^ ^–^ ^340^ ^,^ ^342^ ^–^ ^346^ ^,^ ^348^ ^–^ ^355^ ^,^ ^357^ ^,^ ^358^ ^,^ ^360^ ^–^ ^362^ ^,^ ^364^ ^–^ ^367^ ^,^ ^370^ ^–^ ^377^ ^,^ ^379^ ^–^ ^381^	313 (85.3)
Cross sectional (e.g. survey)	^16^ ^–^ ^23^ ^,^ ^25^ ^,^ ^26^ ^,^ ^28^ ^–^ ^36^ ^,^ ^38^ ^,^ ^40^ ^,^ ^42^ ^–^ ^44^ ^,^ ^46^ ^,^ ^51^ ^,^ ^54^ ^,^ ^57^ ^–^ ^64^ ^,^ ^67^ ^–^ ^72^ ^,^ ^74^ ^–^ ^76^ ^,^ ^79^ ^,^ ^80^ ^,^ ^84^ ^,^ ^85^ ^,^ ^87^ ^–^ ^97^ ^,^ ^100^ ^–^ ^105^ ^,^ ^107^ ^,^ ^109^ ^–^ ^113^ ^,^ ^115^ ^,^ ^116^ ^,^ ^118^ ^,^ ^120^ ^,^ ^123^ ^–^ ^127^ ^,^ ^129^ ^–^ ^132^ ^,^ ^134^ ^,^ ^135^ ^,^ ^138^ ^,^ ^140^ ^,^ ^142^ ^–^ ^144^ ^,^ ^146^ ^,^ ^149^ ^,^ ^150^ ^,^ ^152^ ^,^ ^153^ ^,^ ^155^ ^–^ ^158^ ^,^ ^160^ ^–^ ^165^ ^,^ ^167^ ^,^ ^169^ ^–^ ^171^ ^,^ ^173^ ^,^ ^174^ ^,^ ^178^ ^–^ ^181^ ^,^ ^185^ ^,^ ^187^ ^,^ ^190^ ^,^ ^191^ ^,^ ^193^ ^–^ ^197^ ^,^ ^199^ ^,^ ^202^ ^–^ ^204^ ^,^ ^206^ ^,^ ^208^ ^,^ ^211^ ^–^ ^214^ ^,^ ^217^ ^–^ ^219^ ^,^ ^221^ ^,^ ^224^ ^–^ ^227^ ^,^ ^229^ ^,^ ^230^ ^,^ ^232^ ^,^ ^233^ ^,^ ^237^ ^,^ ^239^ ^,^ ^243^ ^–^ ^245^ ^,^ ^247^ ^,^ ^251^ ^,^ ^252^ ^,^ ^254^ ^–^ ^259^ ^,^ ^261^ ^–^ ^264^ ^,^ ^266^ ^,^ ^269^ ^,^ ^270^ ^,^ ^273^ ^–^ ^275^ ^,^ ^277^ ^–^ ^281^ ^,^ ^283^ ^–^ ^294^ ^,^ ^296^ ^–^ ^298^ ^,^ ^300^ ^–^ ^304^ ^,^ ^306^ ^–^ ^308^ ^,^ ^310^ ^–^ ^312^ ^,^ ^314^ ^–^ ^317^ ^,^ ^320^ ^–^ ^322^ ^,^ ^324^ ^,^ ^327^ ^,^ ^328^ ^,^ ^330^ ^,^ ^332^ ^–^ ^339^ ^,^ ^343^ ^–^ ^345^ ^,^ ^348^ ^–^ ^352^ ^,^ ^354^ ^,^ ^357^ ^,^ ^358^ ^,^ ^360^ ^–^ ^362^ ^,^ ^364^ ^–^ ^367^ ^,^ ^370^ ^–^ ^372^ ^,^ ^374^ ^–^ ^377^ ^,^ ^379^ ^–^ ^381^	257 (70.0)
Cohort (prospective or retrospective)	^81^ ^–^ ^83^ ^,^ ^122^ ^,^ ^128^ ^,^ ^137^ ^,^ ^154^ ^,^ ^159^ ^,^ ^172^ ^,^ ^175^ ^,^ ^182^ ^,^ ^184^ ^,^ ^188^ ^,^ ^198^ ^,^ ^200^ ^,^ ^216^ ^,^ ^220^ ^,^ ^228^ ^,^ ^235^ ^,^ ^238^ ^,^ ^241^ ^,^ ^250^ ^,^ ^268^ ^,^ ^271^ ^,^ ^276^ ^,^ ^282^ ^,^ ^295^ ^,^ ^305^ ^,^ ^309^ ^,^ ^319^ ^,^ ^323^ ^,^ ^325^ ^,^ ^331^ ^,^ ^340^ ^,^ ^346^ ^,^ ^353^ ^,^ ^373^	37 (10.1)
Others (e.g. Delphi or ecological studies)	^15^ ^,^ ^27^ ^,^ ^48^ ^,^ ^56^ ^,^ ^99^ ^,^ ^119^ ^,^ ^132^ ^,^ ^145^ ^,^ ^147^ ^,^ ^151^ ^,^ ^166^ ^,^ ^168^ ^,^ ^177^ ^,^ ^179^ ^,^ ^183^ ^,^ ^223^ ^,^ ^242^ ^,^ ^246^ ^,^ ^342^	19 (5.2)
Secondary research (e.g. review)	^24^ ^,^ ^39^ ^,^ ^41^ ^,^ ^47^ ^,^ ^49^ ^,^ ^53^ ^,^ ^73^ ^,^ ^78^ ^,^ ^86^ ^,^ ^121^ ^,^ ^139^ ^,^ ^148^ ^,^ ^209^ ^,^ ^210^ ^,^ ^222^ ^,^ ^265^ ^,^ ^272^ ^,^ ^326^	18 (4.9)
**Health-care settings**		
Hospital-based or secondary care	^16^ ^,^ ^17^ ^,^ ^20^ ^,^ ^21^ ^,^ ^23^ ^,^ ^26^ ^,^ ^29^ ^–^ ^31^ ^,^ ^33^ ^,^ ^35^ ^–^ ^37^ ^,^ ^39^ ^,^ ^41^ ^,^ ^42^ ^,^ ^46^ ^,^ ^49^ ^,^ ^51^ ^,^ ^55^ ^,^ ^56^ ^,^ ^59^ ^–^ ^62^ ^,^ ^65^ ^,^ ^66^ ^,^ ^71^ ^,^ ^72^ ^,^ ^75^ ^,^ ^76^ ^,^ ^79^ ^–^ ^82^ ^,^ ^84^ ^,^ ^87^ ^–^ ^90^ ^,^ ^93^ ^–^ ^97^ ^,^ ^99^ ^,^ ^101^ ^,^ ^106^ ^,^ ^107^ ^,^ ^109^ ^,^ ^113^ ^,^ ^115^ ^,^ ^125^ ^,^ ^126^ ^,^ ^128^ ^,^ ^130^ ^–^ ^134^ ^,^ ^136^ ^,^ ^137^ ^,^ ^140^ ^,^ ^141^ ^,^ ^143^ ^,^ ^146^ ^,^ ^147^ ^,^ ^150^ ^–^ ^152^ ^,^ ^154^ ^,^ ^155^ ^,^ ^159^ ^,^ ^162^ ^–^ ^165^ ^,^ ^168^ ^,^ ^172^ ^,^ ^175^ ^,^ ^177^ ^,^ ^178^ ^,^ ^181^ ^,^ ^182^ ^,^ ^184^ ^,^ ^185^ ^,^ ^187^ ^–^ ^189^ ^,^ ^191^ ^,^ ^192^ ^,^ ^197^ ^–^ ^200^ ^,^ ^203^ ^,^ ^205^ ^,^ ^207^ ^,^ ^212^ ^,^ ^213^ ^,^ ^215^ ^,^ ^217^ ^–^ ^221^ ^,^ ^223^ ^,^ ^224^ ^,^ ^229^ ^,^ ^230^ ^,^ ^233^ ^–^ ^237^ ^,^ ^239^ ^,^ ^241^ ^,^ ^243^ ^,^ ^244^ ^,^ ^248^ ^,^ ^250^ ^,^ ^253^ ^,^ ^257^ ^,^ ^260^ ^–^ ^263^ ^,^ ^267^ ^,^ ^268^ ^,^ ^270^ ^,^ ^271^ ^,^ ^273^ ^,^ ^274^ ^,^ ^276^ ^,^ ^277^ ^,^ ^282^ ^,^ ^285^ ^,^ ^288^ ^–^ ^290^ ^,^ ^294^ ^,^ ^295^ ^,^ ^297^ ^–^ ^299^ ^,^ ^301^ ^,^ ^302^ ^,^ ^306^ ^,^ ^308^ ^,^ ^310^ ^–^ ^313^ ^,^ ^316^ ^–^ ^319^ ^,^ ^321^ ^,^ ^323^ ^,^ ^325^ ^,^ ^327^ ^–^ ^336^ ^,^ ^338^ ^–^ ^340^ ^,^ ^342^ ^,^ ^343^ ^,^ ^345^ ^,^ ^346^ ^,^ ^348^ ^,^ ^350^ ^,^ ^353^ ^–^ ^356^ ^,^ ^358^ ^,^ ^361^ ^,^ ^365^ ^,^ ^372^ ^,^ ^374^ ^–^ ^381^	195 (53.1)
Home-based or community or primary care	^18^ ^,^ ^19^ ^,^ ^22^ ^,^ ^25^ ^,^ ^27^ ^,^ ^28^ ^,^ ^34^ ^,^ ^38^ ^,^ ^40^ ^,^ ^43^ ^–^ ^45^ ^,^ ^48^ ^,^ ^52^ ^–^ ^54^ ^,^ ^57^ ^,^ ^58^ ^,^ ^64^ ^,^ ^68^ ^,^ ^69^ ^,^ ^73^ ^,^ ^74^ ^,^ ^77^ ^,^ ^86^ ^,^ ^92^ ^,^ ^98^ ^,^ ^100^ ^,^ ^102^ ^–^ ^105^ ^,^ ^108^ ^,^ ^111^ ^,^ ^112^ ^,^ ^114^ ^,^ ^116^ ^–^ ^120^ ^,^ ^122^ ^–^ ^124^ ^,^ ^127^ ^,^ ^129^ ^,^ ^135^ ^,^ ^138^ ^,^ ^144^ ^,^ ^145^ ^,^ ^149^ ^,^ ^153^ ^,^ ^156^ ^–^ ^158^ ^,^ ^160^ ^,^ ^161^ ^,^ ^167^ ^,^ ^169^ ^–^ ^171^ ^,^ ^176^ ^,^ ^180^ ^,^ ^190^ ^,^ ^193^ ^–^ ^196^ ^,^ ^202^ ^,^ ^204^ ^,^ ^206^ ^,^ ^214^ ^,^ ^216^ ^,^ ^225^ ^–^ ^228^ ^,^ ^232^ ^,^ ^238^ ^,^ ^240^ ^,^ ^242^ ^,^ ^245^ ^,^ ^249^ ^,^ ^252^ ^,^ ^254^ ^,^ ^256^ ^,^ ^258^ ^,^ ^259^ ^,^ ^264^ ^–^ ^266^ ^,^ ^269^ ^,^ ^278^ ^–^ ^280^ ^,^ ^283^ ^,^ ^284^ ^,^ ^286^ ^,^ ^291^ ^–^ ^293^ ^,^ ^300^ ^,^ ^303^ ^–^ ^305^ ^,^ ^307^ ^,^ ^309^ ^,^ ^322^ ^,^ ^324^ ^,^ ^326^ ^,^ ^337^ ^,^ ^347^ ^,^ ^351^ ^,^ ^352^ ^,^ ^357^ ^,^ ^359^ ^,^ ^360^ ^,^ ^366^ ^,^ ^368^ ^–^ ^371^ ^,^ ^373^	123 (33.5)
Mixed	^15^ ^,^ ^24^ ^,^ ^47^ ^,^ ^67^ ^,^ ^91^ ^,^ ^110^ ^,^ ^121^ ^,^ ^139^ ^,^ ^142^ ^,^ ^148^ ^,^ ^166^ ^,^ ^183^ ^,^ ^186^ ^,^ ^201^ ^,^ ^210^ ^,^ ^211^ ^,^ ^222^ ^,^ ^247^ ^,^ ^272^ ^,^ ^275^ ^,^ ^281^ ^,^ ^287^ ^,^ ^296^ ^,^ ^344^ ^,^ ^349^ ^,^ ^363^	26 (7.1)
Unclear or not applicable	^32^ ^,^ ^50^ ^,^ ^63^ ^,^ ^70^ ^,^ ^78^ ^,^ ^83^ ^,^ ^85^ ^,^ ^173^ ^,^ ^174^ ^,^ ^179^ ^,^ ^208^ ^,^ ^209^ ^,^ ^231^ ^,^ ^246^ ^,^ ^251^ ^,^ ^255^ ^,^ ^314^ ^,^ ^315^ ^,^ ^320^ ^,^ ^341^ ^,^ ^362^ ^,^ ^364^ ^,^ ^367^	23 (6.3)
**Analysis approach**		
Quantitative	^15^ ^–^ ^17^ ^,^ ^19^ ^–^ ^23^ ^,^ ^25^ ^,^ ^26^ ^,^ ^29^ ^–^ ^32^ ^,^ ^34^ ^–^ ^42^ ^,^ ^44^ ^–^ ^46^ ^,^ ^49^ ^–^ ^51^ ^,^ ^53^ ^–^ ^66^ ^,^ ^70^ ^,^ ^72^ ^–^ ^91^ ^,^ ^93^ ^–^ ^95^ ^,^ ^97^ ^–^ ^104^ ^,^ ^106^ ^–^ ^117^ ^,^ ^120^ ^,^ ^122^ ^–^ ^147^ ^,^ ^149^ ^–^ ^165^ ^,^ ^167^ ^–^ ^170^ ^,^ ^172^ ^–^ ^185^ ^,^ ^187^ ^–^ ^192^ ^,^ ^195^ ^–^ ^200^ ^,^ ^202^ ^–^ ^208^ ^,^ ^211^ ^–^ ^230^ ^,^ ^232^ ^–^ ^243^ ^,^ ^245^ ^,^ ^246^ ^,^ ^248^ ^–^ ^250^ ^,^ ^253^ ^–^ ^255^ ^,^ ^257^ ^–^ ^259^ ^,^ ^261^ ^,^ ^263^ ^,^ ^264^ ^,^ ^266^ ^–^ ^268^ ^,^ ^270^ ^–^ ^274^ ^,^ ^276^ ^–^ ^282^ ^,^ ^284^ ^–^ ^313^ ^,^ ^315^ ^–^ ^331^ ^,^ ^334^ ^,^ ^336^ ^–^ ^338^ ^,^ ^340^ ^–^ ^353^ ^,^ ^355^ ^–^ ^361^ ^,^ ^364^ ^,^ ^365^ ^,^ ^367^ ^–^ ^370^ ^,^ ^372^ ^–^ ^381^	316 (86.1)
Qualitative	^24^ ^,^ ^27^ ^,^ ^33^ ^,^ ^43^ ^,^ ^47^ ^,^ ^48^ ^,^ ^68^ ^,^ ^96^ ^,^ ^118^ ^,^ ^119^ ^,^ ^166^ ^,^ ^193^ ^,^ ^194^ ^,^ ^201^ ^,^ ^244^ ^,^ ^247^ ^,^ ^251^ ^,^ ^256^ ^,^ ^260^ ^,^ ^265^ ^,^ ^269^ ^,^ ^275^ ^,^ ^283^ ^,^ ^332^ ^,^ ^333^ ^,^ ^339^	26 (7.1)
Mixed	^18^ ^,^ ^28^ ^,^ ^52^ ^,^ ^67^ ^,^ ^69^ ^,^ ^71^ ^,^ ^92^ ^,^ ^105^ ^,^ ^121^ ^,^ ^148^ ^,^ ^171^ ^,^ ^186^ ^,^ ^209^ ^,^ ^210^ ^,^ ^231^ ^,^ ^252^ ^,^ ^262^ ^,^ ^314^ ^,^ ^335^ ^,^ ^354^ ^,^ ^357^ ^,^ ^362^ ^,^ ^363^ ^,^ ^366^ ^,^ ^371^	25 (6.8)
**Condition or systems treated^b^**		
Infectious	^15^ ^,^ ^18^ ^,^ ^21^ ^,^ ^24^ ^,^ ^26^ ^,^ ^29^ ^,^ ^30^ ^,^ ^37^ ^,^ ^38^ ^,^ ^43^ ^,^ ^45^ ^,^ ^53^ ^,^ ^55^ ^,^ ^58^ ^,^ ^64^ ^,^ ^67^ ^,^ ^73^ ^,^ ^75^ ^,^ ^87^ ^,^ ^95^ ^,^ ^97^ ^,^ ^101^ ^,^ ^108^ ^,^ ^110^ ^,^ ^114^ ^,^ ^117^ ^,^ ^120^ ^,^ ^127^ ^–^ ^129^ ^,^ ^141^ ^,^ ^144^ ^,^ ^147^ ^,^ ^148^ ^,^ ^154^ ^,^ ^155^ ^,^ ^163^ ^,^ ^170^ ^,^ ^172^ ^,^ ^178^ ^,^ ^181^ ^–^ ^183^ ^,^ ^186^ ^,^ ^187^ ^,^ ^190^ ^,^ ^192^ ^–^ ^194^ ^,^ ^202^ ^,^ ^203^ ^,^ ^207^ ^,^ ^210^ ^,^ ^213^ ^,^ ^220^ ^,^ ^223^ ^,^ ^228^ ^,^ ^230^ ^,^ ^231^ ^,^ ^233^ ^–^ ^235^ ^,^ ^241^ ^,^ ^246^ ^,^ ^249^ ^,^ ^255^ ^,^ ^256^ ^,^ ^260^ ^,^ ^261^ ^,^ ^282^ ^,^ ^284^ ^,^ ^295^ ^,^ ^307^ ^,^ ^314^ ^–^ ^316^ ^,^ ^319^ ^,^ ^327^ ^,^ ^329^ ^,^ ^332^ ^,^ ^339^ ^,^ ^347^ ^,^ ^364^ ^,^ ^367^ ^,^ ^369^ ^,^ ^380^	86 (23.4)
Cardiovascular	^20^ ^,^ ^33^ ^,^ ^36^ ^,^ ^39^ ^,^ ^42^ ^,^ ^46^ ^,^ ^70^ ^,^ ^77^ ^,^ ^103^ ^,^ ^107^ ^,^ ^112^ ^,^ ^132^ ^,^ ^143^ ^,^ ^146^ ^,^ ^157^ ^,^ ^158^ ^,^ ^174^ ^,^ ^184^ ^,^ ^188^ ^,^ ^197^ ^,^ ^204^ ^,^ ^206^ ^,^ ^208^ ^,^ ^217^ ^,^ ^218^ ^,^ ^221^ ^,^ ^236^ ^,^ ^238^ ^,^ ^239^ ^,^ ^241^ ^,^ ^243^ ^,^ ^270^ ^,^ ^284^ ^,^ ^286^ ^,^ ^297^ ^,^ ^300^ ^–^ ^302^ ^,^ ^304^ ^,^ ^305^ ^,^ ^312^ ^,^ ^330^ ^,^ ^336^ ^,^ ^337^ ^,^ ^346^ ^,^ ^358^ ^,^ ^374^ ^,^ ^379^	48 (13.1)
Respiratory	^23^ ^,^ ^56^ ^,^ ^88^ ^,^ ^100^ ^,^ ^103^ ^,^ ^123^ ^,^ ^132^ ^,^ ^143^ ^,^ ^152^ ^,^ ^157^ ^,^ ^179^ ^,^ ^180^ ^,^ ^184^ ^,^ ^204^ ^,^ ^208^ ^,^ ^221^ ^,^ ^257^ ^,^ ^265^ ^,^ ^268^ ^,^ ^273^ ^,^ ^302^ ^,^ ^306^ ^,^ ^309^ ^,^ ^312^ ^,^ ^325^ ^,^ ^330^ ^,^ ^336^ ^,^ ^344^ ^–^ ^346^ ^,^ ^350^ ^,^ ^358^	32 (8.7)
Diabetes	^32^ ^,^ ^33^ ^,^ ^42^ ^,^ ^70^ ^,^ ^77^ ^,^ ^83^ ^,^ ^84^ ^,^ ^103^ ^,^ ^157^ ^,^ ^158^ ^,^ ^174^ ^,^ ^204^ ^,^ ^208^ ^,^ ^217^ ^,^ ^238^ ^,^ ^241^ ^,^ ^248^ ^,^ ^270^ ^,^ ^286^ ^,^ ^300^ ^,^ ^302^ ^,^ ^304^ ^,^ ^310^ ^,^ ^312^ ^,^ ^320^ ^,^ ^321^ ^,^ ^336^ ^,^ ^337^ ^,^ ^342^ ^,^ ^355^ ^,^ ^375^	31 (8.4)
Mental health	^23^ ^,^ ^33^ ^,^ ^35^ ^,^ ^36^ ^,^ ^44^ ^,^ ^54^ ^,^ ^64^ ^,^ ^73^ ^,^ ^75^ ^,^ ^138^ ^,^ ^142^ ^,^ ^195^ ^,^ ^198^ ^,^ ^199^ ^,^ ^213^ ^,^ ^219^ ^,^ ^226^ ^,^ ^232^ ^,^ ^238^ ^,^ ^239^ ^,^ ^253^ ^,^ ^300^ ^,^ ^302^ ^,^ ^305^ ^,^ ^311^ ^,^ ^317^ ^,^ ^337^ ^,^ ^341^ ^,^ ^375^	29 (7.9)
Gastrointestinal	^41^ ^,^ ^64^ ^,^ ^73^ ^,^ ^82^ ^,^ ^122^ ^,^ ^124^ ^,^ ^132^ ^,^ ^143^ ^,^ ^173^ ^,^ ^179^ ^,^ ^188^ ^,^ ^199^ ^,^ ^211^ ^,^ ^212^ ^,^ ^215^ ^,^ ^237^ ^,^ ^243^ ^,^ ^263^ ^,^ ^264^ ^,^ ^287^ ^,^ ^328^ ^,^ ^343^ ^,^ ^353^ ^,^ ^362^	24 (6.5)
Genitourinary	^23^ ^,^ ^36^ ^,^ ^73^ ^,^ ^80^ ^,^ ^93^ ^,^ ^96^ ^,^ ^109^ ^,^ ^132^ ^,^ ^145^ ^,^ ^157^ ^,^ ^198^ ^,^ ^236^ ^,^ ^250^ ^,^ ^323^ ^,^ ^346^ ^,^ ^377^	16 (4.4)
Other (e.g. cancer and skin)	^39^ ^,^ ^42^ ^,^ ^62^ ^,^ ^72^ ^,^ ^115^ ^,^ ^157^ ^,^ ^177^ ^,^ ^185^ ^,^ ^188^ ^,^ ^214^ ^,^ ^221^ ^,^ ^238^ ^,^ ^239^ ^,^ ^262^ ^,^ ^286^ ^,^ ^287^ ^,^ ^302^ ^,^ ^305^ ^,^ ^313^ ^,^ ^329^ ^,^ ^331^ ^,^ ^340^ ^,^ ^348^ ^,^ ^372^	24 (6.5)
Not specified	^16^ ^,^ ^17^ ^,^ ^19^ ^,^ ^22^ ^,^ ^25^ ^,^ ^27^ ^,^ ^28^ ^,^ ^31^ ^,^ ^34^ ^,^ ^40^ ^,^ ^47^ ^–^ ^52^ ^,^ ^57^ ^,^ ^59^ ^–^ ^61^ ^,^ ^63^ ^,^ ^65^ ^,^ ^66^ ^,^ ^68^ ^,^ ^69^ ^,^ ^71^ ^,^ ^74^ ^,^ ^76^ ^,^ ^78^ ^,^ ^79^ ^,^ ^81^ ^,^ ^85^ ^,^ ^86^ ^,^ ^89^ ^–^ ^92^ ^,^ ^94^ ^,^ ^98^ ^,^ ^99^ ^,^ ^102^ ^,^ ^104^ ^–^ ^106^ ^,^ ^111^ ^,^ ^113^ ^,^ ^116^ ^,^ ^118^ ^,^ ^119^ ^,^ ^121^ ^,^ ^125^ ^,^ ^126^ ^,^ ^130^ ^,^ ^131^ ^,^ ^133^ ^–^ ^137^ ^,^ ^139^ ^,^ ^140^ ^,^ ^149^ ^–^ ^151^ ^,^ ^153^ ^,^ ^156^ ^,^ ^159^ ^–^ ^162^ ^,^ ^164^ ^–^ ^171^ ^,^ ^175^ ^,^ ^176^ ^,^ ^189^ ^,^ ^191^ ^,^ ^196^ ^,^ ^200^ ^,^ ^201^ ^,^ ^205^ ^,^ ^209^ ^,^ ^216^ ^,^ ^222^ ^,^ ^224^ ^,^ ^225^ ^,^ ^227^ ^,^ ^229^ ^,^ ^240^ ^,^ ^242^ ^,^ ^244^ ^,^ ^245^ ^,^ ^247^ ^,^ ^251^ ^,^ ^252^ ^,^ ^254^ ^,^ ^258^ ^,^ ^259^ ^,^ ^266^ ^,^ ^267^ ^,^ ^269^ ^,^ ^271^ ^,^ ^272^ ^,^ ^274^ ^–^ ^281^ ^,^ ^283^ ^,^ ^285^ ^,^ ^288^ ^–^ ^294^ ^,^ ^296^ ^,^ ^298^ ^,^ ^299^ ^,^ ^303^ ^,^ ^308^ ^,^ ^318^ ^,^ ^322^ ^,^ ^324^ ^,^ ^326^ ^,^ ^333^ ^–^ ^335^ ^,^ ^338^ ^,^ ^349^ ^,^ ^351^ ^,^ ^352^ ^,^ ^354^ ^,^ ^356^ ^,^ ^357^ ^,^ ^359^ ^–^ ^361^ ^,^ ^363^ ^,^ ^365^ ^,^ ^366^ ^,^ ^368^ ^,^ ^370^ ^,^ ^371^ ^,^ ^373^ ^,^ ^376^ ^,^ ^378^ ^,^ ^381^	156 (42.5)
**Medication class^b^**		
Antimicrobials	^15^ ^,^ ^17^ ^,^ ^18^ ^,^ ^21^ ^–^ ^30^ ^,^ ^38^ ^,^ ^45^ ^,^ ^48^ ^–^ ^50^ ^,^ ^52^ ^,^ ^53^ ^,^ ^55^ ^,^ ^56^ ^,^ ^58^ ^,^ ^61^ ^,^ ^64^ ^,^ ^65^ ^,^ ^67^ ^–^ ^71^ ^,^ ^73^ ^,^ ^75^ ^,^ ^80^ ^,^ ^81^ ^,^ ^84^ ^,^ ^87^ ^,^ ^88^ ^,^ ^91^ ^,^ ^95^ ^–^ ^101^ ^,^ ^103^ ^,^ ^108^ ^,^ ^110^ ^,^ ^111^ ^,^ ^113^ ^,^ ^114^ ^,^ ^117^ ^–^ ^129^ ^,^ ^135^ ^,^ ^141^ ^,^ ^144^ ^,^ ^147^ ^,^ ^149^ ^,^ ^156^ ^,^ ^163^ ^,^ ^167^ ^–^ ^169^ ^,^ ^171^ ^,^ ^175^ ^,^ ^179^ ^,^ ^185^ ^,^ ^187^ ^,^ ^192^ ^,^ ^202^ ^,^ ^203^ ^,^ ^205^ ^,^ ^209^ ^,^ ^222^ ^,^ ^223^ ^,^ ^228^ ^,^ ^230^ ^,^ ^231^ ^,^ ^234^ ^–^ ^236^ ^,^ ^245^ ^–^ ^247^ ^,^ ^249^ ^,^ ^251^ ^,^ ^252^ ^,^ ^255^ ^,^ ^256^ ^,^ ^260^ ^,^ ^263^ ^–^ ^265^ ^,^ ^269^ ^,^ ^272^ ^,^ ^274^ ^,^ ^279^ ^,^ ^282^ ^,^ ^283^ ^,^ ^285^ ^,^ ^287^ ^,^ ^288^ ^,^ ^290^ ^,^ ^293^ ^–^ ^296^ ^,^ ^298^ ^–^ ^300^ ^,^ ^307^ ^,^ ^309^ ^,^ ^313^ ^–^ ^316^ ^,^ ^319^ ^,^ ^326^ ^–^ ^328^ ^,^ ^331^ ^,^ ^332^ ^,^ ^339^ ^,^ ^343^ ^,^ ^347^ ^,^ ^350^ ^,^ ^353^ ^,^ ^357^ ^,^ ^360^ ^,^ ^362^ ^–^ ^364^ ^,^ ^366^ ^–^ ^369^ ^,^ ^371^ ^,^ ^376^ ^,^ ^380^ ^,^ ^381^	152 (41.4)
Psychotropic drugs^c^	^19^ ^,^ ^31^ ^,^ ^34^ ^–^ ^37^ ^,^ ^40^ ^,^ ^42^ ^,^ ^44^ ^,^ ^54^ ^,^ ^57^ ^,^ ^60^ ^,^ ^63^ ^,^ ^70^ ^,^ ^92^ ^,^ ^102^ ^,^ ^104^ ^,^ ^106^ ^,^ ^107^ ^,^ ^116^ ^,^ ^131^ ^–^ ^133^ ^,^ ^136^ ^–^ ^138^ ^,^ ^142^ ^,^ ^143^ ^,^ ^157^ ^,^ ^158^ ^,^ ^160^ ^,^ ^161^ ^,^ ^168^ ^,^ ^169^ ^,^ ^174^ ^,^ ^176^ ^,^ ^178^ ^,^ ^181^ ^,^ ^182^ ^,^ ^184^ ^,^ ^188^ ^,^ ^196^ ^,^ ^198^ ^,^ ^199^ ^,^ ^204^ ^,^ ^206^ ^,^ ^208^ ^,^ ^213^ ^,^ ^216^ ^–^ ^219^ ^,^ ^221^ ^,^ ^225^ ^,^ ^227^ ^,^ ^232^ ^,^ ^238^ ^,^ ^239^ ^,^ ^241^ ^,^ ^243^ ^,^ ^248^ ^,^ ^250^ ^,^ ^253^ ^,^ ^266^ ^,^ ^270^ ^–^ ^272^ ^,^ ^276^ ^,^ ^286^ ^,^ ^289^ ^,^ ^297^ ^,^ ^300^ ^,^ ^302^ ^,^ ^304^ ^,^ ^305^ ^,^ ^311^ ^,^ ^312^ ^,^ ^317^ ^,^ ^318^ ^,^ ^330^ ^,^ ^336^ ^,^ ^348^ ^,^ ^349^ ^,^ ^352^ ^,^ ^354^ ^,^ ^356^ ^,^ ^358^ ^,^ ^361^ ^,^ ^374^ ^,^ ^375^ ^,^ ^379^	91 (24.8)
Blood pressure lowering and cardiovascular medications	^20^ ^,^ ^25^ ^,^ ^33^ ^,^ ^34^ ^,^ ^36^ ^,^ ^39^ ^,^ ^42^ ^,^ ^44^ ^,^ ^54^ ^,^ ^77^ ^,^ ^80^ ^,^ ^89^ ^,^ ^92^ ^,^ ^104^ ^,^ ^107^ ^,^ ^112^ ^,^ ^132^ ^,^ ^138^ ^,^ ^143^ ^,^ ^146^ ^,^ ^150^ ^,^ ^158^ ^,^ ^160^ ^–^ ^162^ ^,^ ^165^ ^,^ ^169^ ^,^ ^182^ ^,^ ^188^ ^,^ ^196^ ^,^ ^197^ ^,^ ^199^ ^,^ ^204^ ^,^ ^206^ ^,^ ^208^ ^,^ ^213^ ^,^ ^216^ ^–^ ^218^ ^,^ ^221^ ^,^ ^225^ ^,^ ^227^ ^,^ ^229^ ^,^ ^236^ ^,^ ^238^ ^,^ ^239^ ^,^ ^243^ ^,^ ^248^ ^,^ ^250^ ^,^ ^258^ ^,^ ^266^ ^,^ ^272^ ^,^ ^277^ ^,^ ^291^ ^,^ ^297^ ^,^ ^300^ ^,^ ^302^ ^,^ ^305^ ^,^ ^317^ ^,^ ^320^ ^–^ ^322^ ^,^ ^324^ ^,^ ^330^ ^,^ ^334^ ^,^ ^337^ ^,^ ^338^ ^,^ ^341^ ^,^ ^346^ ^,^ ^349^ ^,^ ^352^ ^,^ ^353^ ^,^ ^370^ ^,^ ^374^ ^,^ ^375^ ^,^ ^379^	76 (20.7)
Analgesics and steroids	^19^ ^,^ ^25^ ^,^ ^31^ ^,^ ^34^ ^,^ ^36^ ^,^ ^39^ ^,^ ^40^ ^,^ ^43^ ^,^ ^44^ ^,^ ^60^ ^,^ ^67^ ^,^ ^70^ ^,^ ^80^ ^,^ ^88^ ^,^ ^96^ ^,^ ^104^ ^,^ ^106^ ^,^ ^116^ ^,^ ^131^ ^,^ ^138^ ^,^ ^140^ ^,^ ^143^ ^,^ ^146^ ^,^ ^150^ ^,^ ^152^ ^,^ ^159^ ^,^ ^169^ ^,^ ^176^ ^,^ ^180^ ^–^ ^182^ ^,^ ^184^ ^,^ ^188^ ^,^ ^195^ ^,^ ^196^ ^,^ ^198^ ^,^ ^204^ ^,^ ^213^ ^,^ ^226^ ^,^ ^229^ ^,^ ^236^ ^,^ ^238^ ^,^ ^239^ ^,^ ^246^ ^,^ ^250^ ^,^ ^257^ ^,^ ^272^ ^,^ ^273^ ^,^ ^276^ ^,^ ^277^ ^,^ ^286^ ^,^ ^291^ ^,^ ^297^ ^,^ ^300^ ^,^ ^302^ ^–^ ^304^ ^,^ ^310^ ^,^ ^317^ ^,^ ^325^ ^,^ ^330^ ^,^ ^336^ ^,^ ^337^ ^,^ ^340^ ^,^ ^344^ ^,^ ^346^ ^,^ ^349^ ^,^ ^352^ ^–^ ^354^ ^,^ ^375^	71 (19.3)
Proton pump inhibitors and antacids	^33^ ^,^ ^39^ ^–^ ^42^ ^,^ ^44^ ^,^ ^79^ ^,^ ^82^ ^,^ ^89^ ^,^ ^90^ ^,^ ^94^ ^,^ ^96^ ^,^ ^107^ ^,^ ^109^ ^,^ ^132^ ^,^ ^134^ ^,^ ^165^ ^,^ ^173^ ^,^ ^176^ ^,^ ^178^ ^,^ ^188^ ^,^ ^196^ ^,^ ^200^ ^,^ ^204^ ^,^ ^212^ ^,^ ^215^ ^,^ ^218^ ^,^ ^221^ ^,^ ^225^ ^,^ ^227^ ^,^ ^236^ ^,^ ^237^ ^,^ ^241^ ^,^ ^246^ ^,^ ^248^ ^,^ ^266^ ^,^ ^276^ ^,^ ^277^ ^,^ ^280^ ^,^ ^286^ ^,^ ^303^ ^,^ ^310^ ^,^ ^312^ ^,^ ^336^ ^,^ ^346^ ^,^ ^352^ ^,^ ^353^ ^,^ ^358^ ^,^ ^361^ ^,^ ^365^ ^,^ ^374^ ^,^ ^375^ ^,^ ^378^	53 (14.4)
Hypoglycaemic and hormonal medications	^32^ ^,^ ^34^ ^,^ ^39^ ^,^ ^44^ ^,^ ^77^ ^,^ ^80^ ^,^ ^83^ ^,^ ^103^ ^,^ ^112^ ^,^ ^143^ ^,^ ^158^ ^,^ ^160^ ^,^ ^161^ ^,^ ^169^ ^,^ ^206^ ^,^ ^208^ ^,^ ^216^ ^,^ ^217^ ^,^ ^248^ ^,^ ^266^ ^,^ ^300^ ^,^ ^304^ ^,^ ^312^ ^,^ ^320^ ^,^ ^321^ ^,^ ^342^ ^,^ ^349^ ^,^ ^355^ ^,^ ^375^ ^,^ ^376^	30 (8.2)
Others	^16^ ^,^ ^25^ ^,^ ^72^ ^,^ ^74^ ^,^ ^85^ ^,^ ^115^ ^,^ ^162^ ^,^ ^214^ ^,^ ^246^ ^,^ ^258^ ^,^ ^262^ ^,^ ^322^ ^,^ ^324^ ^,^ ^329^ ^,^ ^338^ ^,^ ^370^ ^,^ ^372^ ^,^ ^377^	18 (4.9)
Not specified	^46^ ^,^ ^47^ ^,^ ^51^ ^,^ ^59^ ^,^ ^62^ ^,^ ^66^ ^,^ ^76^ ^,^ ^78^ ^,^ ^86^ ^,^ ^93^ ^,^ ^105^ ^,^ ^130^ ^,^ ^139^ ^,^ ^145^ ^,^ ^148^ ^,^ ^151^ ^,^ ^153^ ^–^ ^155^ ^,^ ^164^ ^,^ ^166^ ^,^ ^170^ ^,^ ^172^ ^,^ ^177^ ^,^ ^183^ ^,^ ^186^ ^,^ ^189^ ^–^ ^191^ ^,^ ^193^ ^,^ ^194^ ^,^ ^201^ ^,^ ^207^ ^,^ ^210^ ^,^ ^211^ ^,^ ^220^ ^,^ ^224^ ^,^ ^233^ ^,^ ^240^ ^,^ ^242^ ^,^ ^244^ ^,^ ^254^ ^,^ ^259^ ^,^ ^261^ ^,^ ^267^ ^,^ ^268^ ^,^ ^275^ ^,^ ^278^ ^,^ ^281^ ^,^ ^284^ ^,^ ^292^ ^,^ ^301^ ^,^ ^306^ ^,^ ^308^ ^,^ ^323^ ^,^ ^333^ ^,^ ^335^ ^,^ ^345^ ^,^ ^351^ ^,^ ^359^ ^,^ ^373^	61 (16.6)

RCT: randomized controlled trial.

^a^ Twenty-one studies covered more than one region.

^b^ Not mutually exclusive.

^c^ Including anti-anxiety, antidepressant, anticonvulsant and antipsychotic.

Most studies (330; 89.9%) were published after 2010, with a marked increase in the number of studies per year. Most studies were written in English (347; 94.6%). In 195 studies (53.1%), the health-care setting was hospital-based. The study design in 313 (85.3%) studies was observational and in 26 (7.1%) studies was qualitative only. Twelve studies (3.3%) declared any funding from industry sources (Table 1; data repository).^14^

### Extent of overuse

**Fig. 3:**
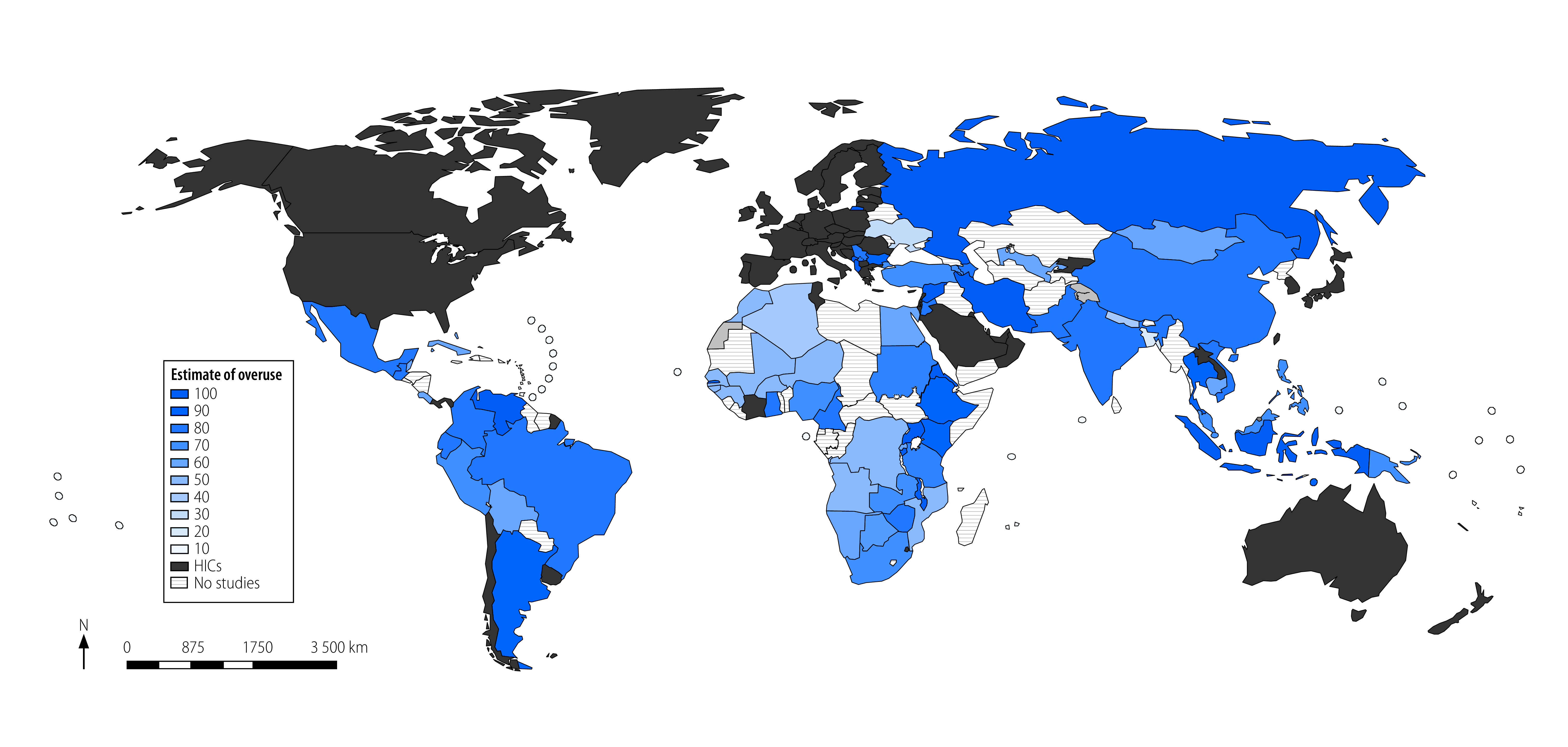
Average percentage of overuse of medications in low- and middle-income countries

Note: We obtained the data from studies reporting on the extent of overuse of medications.

Of the included studies, 307 (83.7%; Table 2) reported on the extent of overuse of medications. Overall, the estimates of overuse of medications ranged from 7.3% in a study of 1264 older adults with diabetes in Türkiye (i.e. overtreated with blood pressure lowering and anti-diabetic medications)^320^ to 98.2% in a study of 599 critically ill older patients (i.e. ≥ 1 potentially inappropriate medications).^141^ The IQR of the estimates reported in 307 studies was 30.2–64.5% (Box 2; Fig. 3).

**Table 2:** Studies included on the extent of overuse of medications in low- and middle-income countries

Study characteristic	Study reference	No. (%) of studies
**Total**	^15^ ^,^ ^16^ ^,^ ^19^ ^–^ ^26^ ^,^ ^28^ ^,^ ^30^ ^–^ ^42^ ^,^ ^44^ ^–^ ^46^ ^,^ ^49^ ^–^ ^51^ ^,^ ^53^ ^,^ ^54^ ^,^ ^56^ ^–^ ^64^ ^,^ ^66^ ^,^ ^67^ ^,^ ^70^ ^–^ ^97^ ^,^ ^100^ ^–^ ^102^ ^,^ ^104^ ^–^ ^107^ ^,^ ^109^ ^–^ ^113^ ^,^ ^115^ ^,^ ^116^ ^,^ ^120^ ^–^ ^154^ ^,^ ^157^ ^–^ ^163^ ^,^ ^165^ ^,^ ^167^ ^–^ ^175^ ^,^ ^177^ ^–^ ^191^ ^,^ ^195^ ^–^ ^208^ ^,^ ^210^ ^–^ ^214^ ^,^ ^216^ ^–^ ^230^ ^,^ ^232^ ^–^ ^235^ ^,^ ^237^ ^–^ ^243^ ^,^ ^245^ ^,^ ^248^ ^–^ ^250^ ^,^ ^252^ ^–^ ^259^ ^,^ ^261^ ^–^ ^264^ ^,^ ^266^ ^–^ ^273^ ^,^ ^276^ ^–^ ^282^ ^,^ ^284^ ^–^ ^298^ ^,^ ^300^ ^–^ ^312^ ^,^ ^314^ ^–^ ^328^ ^,^ ^330^ ^,^ ^332^ ^,^ ^334^ ^,^ ^336^ ^–^ ^338^ ^,^ ^340^ ^,^ ^342^ ^–^ ^346^ ^,^ ^348^ ^–^ ^355^ ^,^ ^358^ ^,^ ^360^ ^–^ ^362^ ^,^ ^364^ ^–^ ^368^ ^,^ ^370^ ^,^ ^372^ ^–^ ^377^ ^,^ ^379^	307 (83.7)^a^
**Country income level^b^**		
Low income	^20^ ^,^ ^21^ ^,^ ^38^ ^,^ ^67^ ^,^ ^75^ ^,^ ^77^ ^,^ ^111^ ^,^ ^127^ ^,^ ^129^ ^,^ ^144^ ^,^ ^146^ ^,^ ^170^ ^,^ ^183^ ^,^ ^230^ ^,^ ^245^ ^,^ ^249^ ^,^ ^256^ ^,^ ^262^ ^,^ ^316^ ^,^ ^337^ ^,^ ^343^	21 (6.8)
Lower-middle income	^16^ ^,^ ^23^ ^,^ ^25^ ^,^ ^28^ ^,^ ^31^ ^,^ ^45^ ^,^ ^61^ ^,^ ^64^ ^,^ ^71^ ^,^ ^72^ ^,^ ^74^ ^,^ ^78^ ^,^ ^79^ ^,^ ^81^ ^,^ ^85^ ^,^ ^93^ ^,^ ^122^ ^,^ ^124^ ^,^ ^130^ ^,^ ^131^ ^,^ ^134^ ^,^ ^145^ ^,^ ^150^ ^,^ ^159^ ^,^ ^167^ ^,^ ^168^ ^,^ ^175^ ^,^ ^179^ ^,^ ^181^ ^,^ ^182^ ^,^ ^185^ ^,^ ^186^ ^,^ ^191^ ^,^ ^197^ ^,^ ^220^ ^,^ ^226^ ^,^ ^229^ ^,^ ^234^ ^,^ ^235^ ^,^ ^240^ ^,^ ^241^ ^,^ ^252^ ^,^ ^253^ ^,^ ^261^ ^,^ ^263^ ^,^ ^281^ ^,^ ^284^ ^,^ ^287^ ^,^ ^288^ ^,^ ^293^ ^,^ ^295^ ^,^ ^298^ ^,^ ^301^ ^,^ ^302^ ^,^ ^307^ ^,^ ^308^ ^,^ ^310^ ^–^ ^312^ ^,^ ^315^ ^,^ ^319^ ^,^ ^346^ ^,^ ^353^	63 (20.5)
Upper-middle income	^15^ ^,^ ^19^ ^,^ ^22^ ^,^ ^26^ ^,^ ^30^ ^,^ ^32^ ^–^ ^37^ ^,^ ^39^ ^–^ ^42^ ^,^ ^44^ ^,^ ^46^ ^,^ ^49^ ^–^ ^51^ ^,^ ^54^ ^,^ ^56^ ^,^ ^57^ ^,^ ^59^ ^,^ ^60^ ^,^ ^62^ ^,^ ^63^ ^,^ ^66^ ^,^ ^70^ ^,^ ^76^ ^,^ ^80^ ^,^ ^82^ ^,^ ^84^ ^,^ ^87^ ^–^ ^90^ ^,^ ^92^ ^,^ ^94^ ^–^ ^97^ ^,^ ^100^ ^–^ ^102^ ^,^ ^104^ ^–^ ^107^ ^,^ ^109^ ^,^ ^110^ ^,^ ^112^ ^,^ ^113^ ^,^ ^115^ ^,^ ^116^ ^,^ ^120^ ^,^ ^121^ ^,^ ^123^ ^,^ ^125^ ^,^ ^126^ ^,^ ^128^ ^,^ ^132^ ^,^ ^133^ ^,^ ^135^ ^–^ ^138^ ^,^ ^141^ ^–^ ^143^ ^,^ ^147^ ^,^ ^149^ ^,^ ^151^ ^–^ ^154^ ^,^ ^157^ ^,^ ^158^ ^,^ ^160^ ^–^ ^163^ ^,^ ^165^ ^,^ ^169^ ^,^ ^171^ ^–^ ^174^ ^,^ ^177^ ^,^ ^178^ ^,^ ^184^ ^,^ ^187^ ^–^ ^190^ ^,^ ^195^ ^,^ ^196^ ^,^ ^198^ ^–^ ^200^ ^,^ ^202^ ^–^ ^208^ ^,^ ^211^ ^–^ ^214^ ^,^ ^216^ ^–^ ^219^ ^,^ ^221^ ^,^ ^223^ ^–^ ^225^ ^,^ ^227^ ^,^ ^228^ ^,^ ^232^ ^,^ ^233^ ^,^ ^237^ ^–^ ^239^ ^,^ ^243^ ^,^ ^248^ ^,^ ^250^ ^,^ ^254^ ^,^ ^255^ ^,^ ^257^ ^–^ ^259^ ^,^ ^264^ ^,^ ^266^ ^–^ ^270^ ^,^ ^272^ ^,^ ^273^ ^,^ ^276^ ^–^ ^280^ ^,^ ^282^ ^,^ ^285^ ^,^ ^286^ ^,^ ^289^ ^–^ ^292^ ^,^ ^294^ ^,^ ^296^ ^,^ ^300^ ^,^ ^303^ ^–^ ^306^ ^,^ ^309^ ^,^ ^314^ ^,^ ^317^ ^,^ ^318^ ^,^ ^320^ ^–^ ^325^ ^,^ ^327^ ^,^ ^328^ ^,^ ^330^ ^,^ ^334^ ^,^ ^336^ ^,^ ^338^ ^,^ ^340^ ^,^ ^342^ ^,^ ^344^ ^,^ ^345^ ^,^ ^348^ ^–^ ^352^ ^,^ ^354^ ^,^ ^355^ ^,^ ^358^ ^,^ ^360^ ^–^ ^362^ ^,^ ^364^ ^–^ ^368^ ^,^ ^370^ ^,^ ^372^ ^–^ ^377^ ^,^ ^379^	205 (66.8)
**Medication class**		
Antimicrobials	^15^ ^,^ ^21^ ^–^ ^26^ ^,^ ^28^ ^,^ ^30^ ^,^ ^38^ ^,^ ^45^ ^,^ ^49^ ^,^ ^50^ ^,^ ^53^ ^,^ ^56^ ^,^ ^58^ ^,^ ^61^ ^,^ ^64^ ^,^ ^67^ ^,^ ^70^ ^,^ ^71^ ^,^ ^73^ ^,^ ^75^ ^,^ ^80^ ^,^ ^81^ ^,^ ^84^ ^,^ ^87^ ^,^ ^88^ ^,^ ^95^ ^–^ ^97^ ^,^ ^100^ ^,^ ^101^ ^,^ ^110^ ^,^ ^111^ ^,^ ^113^ ^,^ ^120^ ^–^ ^129^ ^,^ ^135^ ^,^ ^141^ ^,^ ^144^ ^,^ ^147^ ^,^ ^149^ ^,^ ^163^ ^,^ ^167^ ^–^ ^169^ ^,^ ^171^ ^,^ ^175^ ^,^ ^179^ ^,^ ^185^ ^,^ ^187^ ^,^ ^202^ ^,^ ^203^ ^,^ ^205^ ^,^ ^222^ ^,^ ^223^ ^,^ ^228^ ^,^ ^230^ ^,^ ^234^ ^,^ ^235^ ^,^ ^245^ ^,^ ^249^ ^,^ ^252^ ^,^ ^255^ ^,^ ^256^ ^,^ ^263^ ^,^ ^264^ ^,^ ^269^ ^,^ ^272^ ^,^ ^279^ ^,^ ^282^ ^,^ ^285^ ^,^ ^287^ ^,^ ^288^ ^,^ ^290^ ^,^ ^293^ ^–^ ^296^ ^,^ ^298^ ^,^ ^300^ ^,^ ^307^ ^,^ ^309^ ^,^ ^314^ ^–^ ^316^ ^,^ ^319^ ^,^ ^326^ ^–^ ^328^ ^,^ ^332^ ^,^ ^343^ ^,^ ^350^ ^,^ ^353^ ^,^ ^360^ ^,^ ^362^ ^,^ ^364^ ^,^ ^366^ ^–^ ^368^ ^,^ ^376^	110 (35.8)
Low-income country	^21^ ^,^ ^38^ ^,^ ^67^ ^,^ ^75^ ^,^ ^111^ ^,^ ^127^ ^,^ ^129^ ^,^ ^144^ ^,^ ^230^ ^,^ ^245^ ^,^ ^249^ ^,^ ^256^ ^,^ ^316^ ^,^ ^343^	14 (12.7)
Lower-middle-income country	^23^ ^,^ ^25^ ^,^ ^28^ ^,^ ^45^ ^,^ ^61^ ^,^ ^64^ ^,^ ^71^ ^,^ ^81^ ^,^ ^122^ ^,^ ^124^ ^,^ ^167^ ^,^ ^168^ ^,^ ^175^ ^,^ ^179^ ^,^ ^185^ ^,^ ^234^ ^,^ ^235^ ^,^ ^252^ ^,^ ^263^ ^,^ ^287^ ^,^ ^288^ ^,^ ^293^ ^,^ ^295^ ^,^ ^298^ ^,^ ^307^ ^,^ ^315^ ^,^ ^319^ ^,^ ^353^	28 (25.5)
Upper-middle income country	^15^ ^,^ ^22^ ^,^ ^26^ ^,^ ^30^ ^,^ ^49^ ^,^ ^50^ ^,^ ^56^ ^,^ ^70^ ^,^ ^80^ ^,^ ^84^ ^,^ ^87^ ^,^ ^88^ ^,^ ^95^ ^–^ ^97^ ^,^ ^100^ ^,^ ^101^ ^,^ ^110^ ^,^ ^113^ ^,^ ^120^ ^,^ ^121^ ^,^ ^123^ ^,^ ^125^ ^,^ ^126^ ^,^ ^128^ ^,^ ^135^ ^,^ ^141^ ^,^ ^147^ ^,^ ^149^ ^,^ ^163^ ^,^ ^169^ ^,^ ^171^ ^,^ ^187^ ^,^ ^202^ ^,^ ^203^ ^,^ ^205^ ^,^ ^223^ ^,^ ^228^ ^,^ ^255^ ^,^ ^264^ ^,^ ^269^ ^,^ ^272^ ^,^ ^279^ ^,^ ^282^ ^,^ ^285^ ^,^ ^290^ ^,^ ^294^ ^,^ ^296^ ^,^ ^300^ ^,^ ^309^ ^,^ ^314^ ^,^ ^327^ ^,^ ^328^ ^,^ ^350^ ^,^ ^360^ ^,^ ^362^ ^,^ ^364^ ^,^ ^366^ ^–^ ^368^ ^,^ ^376^	61 (55.5)
Psychotropic drugs^c^	^19^ ^,^ ^31^ ^,^ ^34^ ^–^ ^37^ ^,^ ^40^ ^,^ ^42^ ^,^ ^44^ ^,^ ^54^ ^,^ ^57^ ^,^ ^60^ ^,^ ^63^ ^,^ ^70^ ^,^ ^92^ ^,^ ^102^ ^,^ ^104^ ^,^ ^106^ ^,^ ^107^ ^,^ ^116^ ^,^ ^131^ ^–^ ^133^ ^,^ ^136^ ^–^ ^138^ ^,^ ^142^ ^,^ ^143^ ^,^ ^157^ ^,^ ^158^ ^,^ ^160^ ^,^ ^161^ ^,^ ^168^ ^,^ ^169^ ^,^ ^174^ ^,^ ^178^ ^,^ ^181^ ^,^ ^182^ ^,^ ^184^ ^,^ ^188^ ^,^ ^196^ ^,^ ^198^ ^,^ ^199^ ^,^ ^204^ ^,^ ^206^ ^,^ ^208^ ^,^ ^213^ ^,^ ^216^ ^–^ ^219^ ^,^ ^221^ ^,^ ^225^ ^,^ ^227^ ^,^ ^232^ ^,^ ^238^ ^,^ ^239^ ^,^ ^241^ ^,^ ^243^ ^,^ ^248^ ^,^ ^250^ ^,^ ^253^ ^,^ ^266^ ^,^ ^270^ ^–^ ^272^ ^,^ ^276^ ^,^ ^286^ ^,^ ^289^ ^,^ ^297^ ^,^ ^300^ ^,^ ^302^ ^,^ ^304^ ^,^ ^305^ ^,^ ^311^ ^,^ ^312^ ^,^ ^317^ ^,^ ^318^ ^,^ ^330^ ^,^ ^336^ ^,^ ^348^ ^,^ ^349^ ^,^ ^352^ ^,^ ^354^ ^,^ ^358^ ^,^ ^361^ ^,^ ^374^ ^,^ ^375^ ^,^ ^379^	89 (30.0)
Low-income country	N/A	0 (0.0)
Lower-middle income country	^31^ ^,^ ^131^ ^,^ ^168^ ^,^ ^181^ ^,^ ^182^ ^,^ ^241^ ^,^ ^253^ ^,^ ^302^ ^,^ ^311^ ^,^ ^312^	10 (11.2)
Upper-middle income country	^19^ ^,^ ^34^ ^–^ ^37^ ^,^ ^40^ ^,^ ^42^ ^,^ ^44^ ^,^ ^54^ ^,^ ^57^ ^,^ ^60^ ^,^ ^63^ ^,^ ^70^ ^,^ ^92^ ^,^ ^102^ ^,^ ^104^ ^,^ ^106^ ^,^ ^107^ ^,^ ^116^ ^,^ ^132^ ^,^ ^133^ ^,^ ^136^ ^–^ ^138^ ^,^ ^142^ ^,^ ^143^ ^,^ ^157^ ^,^ ^158^ ^,^ ^160^ ^,^ ^161^ ^,^ ^169^ ^,^ ^174^ ^,^ ^178^ ^,^ ^184^ ^,^ ^188^ ^,^ ^196^ ^,^ ^198^ ^,^ ^199^ ^,^ ^204^ ^,^ ^206^ ^,^ ^208^ ^,^ ^213^ ^,^ ^216^ ^–^ ^219^ ^,^ ^221^ ^,^ ^225^ ^,^ ^227^ ^,^ ^232^ ^,^ ^238^ ^,^ ^239^ ^,^ ^243^ ^,^ ^248^ ^,^ ^250^ ^,^ ^266^ ^,^ ^270^ ^,^ ^272^ ^,^ ^276^ ^,^ ^286^ ^,^ ^289^ ^,^ ^300^ ^,^ ^304^ ^,^ ^305^ ^,^ ^317^ ^,^ ^318^ ^,^ ^330^ ^,^ ^336^ ^,^ ^348^ ^,^ ^349^ ^,^ ^352^ ^,^ ^354^ ^,^ ^358^ ^,^ ^361^ ^,^ ^374^ ^,^ ^375^ ^,^ ^379^	77 (86.5)
Antihypertensive	^20^ ^,^ ^25^ ^,^ ^33^ ^,^ ^34^ ^,^ ^36^ ^,^ ^39^ ^,^ ^42^ ^,^ ^44^ ^,^ ^54^ ^,^ ^77^ ^,^ ^80^ ^,^ ^89^ ^,^ ^92^ ^,^ ^104^ ^,^ ^107^ ^,^ ^112^ ^,^ ^132^ ^,^ ^138^ ^,^ ^143^ ^,^ ^146^ ^,^ ^150^ ^,^ ^158^ ^,^ ^160^ ^–^ ^162^ ^,^ ^165^ ^,^ ^169^ ^,^ ^182^ ^,^ ^188^ ^,^ ^196^ ^,^ ^197^ ^,^ ^199^ ^,^ ^204^ ^,^ ^206^ ^,^ ^208^ ^,^ ^213^ ^,^ ^216^ ^–^ ^218^ ^,^ ^221^ ^,^ ^225^ ^,^ ^227^ ^,^ ^229^ ^,^ ^238^ ^,^ ^239^ ^,^ ^243^ ^,^ ^248^ ^,^ ^250^ ^,^ ^258^ ^,^ ^266^ ^,^ ^272^ ^,^ ^277^ ^,^ ^291^ ^,^ ^297^ ^,^ ^300^ ^,^ ^302^ ^,^ ^305^ ^,^ ^317^ ^,^ ^320^ ^–^ ^322^ ^,^ ^324^ ^,^ ^330^ ^,^ ^334^ ^,^ ^337^ ^,^ ^338^ ^,^ ^346^ ^,^ ^349^ ^,^ ^352^ ^,^ ^353^ ^,^ ^370^ ^,^ ^374^ ^,^ ^375^ ^,^ ^379^	74 (24.1)
Analgesics and steroids	^19^ ^,^ ^25^ ^,^ ^31^ ^,^ ^34^ ^,^ ^36^ ^,^ ^39^ ^,^ ^40^ ^,^ ^44^ ^,^ ^60^ ^,^ ^67^ ^,^ ^70^ ^,^ ^80^ ^,^ ^88^ ^,^ ^96^ ^,^ ^104^ ^,^ ^106^ ^,^ ^116^ ^,^ ^131^ ^,^ ^138^ ^,^ ^140^ ^,^ ^143^ ^,^ ^146^ ^,^ ^150^ ^,^ ^152^ ^,^ ^159^ ^,^ ^169^ ^,^ ^180^ ^–^ ^182^ ^,^ ^184^ ^,^ ^188^ ^,^ ^195^ ^,^ ^196^ ^,^ ^198^ ^,^ ^204^ ^,^ ^213^ ^,^ ^226^ ^,^ ^229^ ^,^ ^238^ ^,^ ^239^ ^,^ ^250^ ^,^ ^257^ ^,^ ^272^ ^,^ ^273^ ^,^ ^276^ ^,^ ^277^ ^,^ ^286^ ^,^ ^291^ ^,^ ^297^ ^,^ ^300^ ^,^ ^302^ ^–^ ^304^ ^,^ ^310^ ^,^ ^317^ ^,^ ^325^ ^,^ ^330^ ^,^ ^336^ ^,^ ^337^ ^,^ ^340^ ^,^ ^344^ ^,^ ^346^ ^,^ ^349^ ^,^ ^352^ ^–^ ^354^ ^,^ ^375^	67 (21.8)
Proton pump inhibitors and antacids	^33^ ^,^ ^39^ ^–^ ^42^ ^,^ ^44^ ^,^ ^79^ ^,^ ^82^ ^,^ ^89^ ^,^ ^90^ ^,^ ^94^ ^,^ ^96^ ^,^ ^107^ ^,^ ^109^ ^,^ ^132^ ^,^ ^134^ ^,^ ^165^ ^,^ ^173^ ^,^ ^178^ ^,^ ^188^ ^,^ ^196^ ^,^ ^200^ ^,^ ^204^ ^,^ ^212^ ^,^ ^218^ ^,^ ^221^ ^,^ ^225^ ^,^ ^227^ ^,^ ^237^ ^,^ ^241^ ^,^ ^248^ ^,^ ^266^ ^,^ ^276^ ^,^ ^277^ ^,^ ^280^ ^,^ ^286^ ^,^ ^303^ ^,^ ^310^ ^,^ ^312^ ^,^ ^336^ ^,^ ^346^ ^,^ ^352^ ^,^ ^353^ ^,^ ^358^ ^,^ ^361^ ^,^ ^365^ ^,^ ^374^ ^,^ ^375^	48 (15.6)
**Methods for assessing overuse**		
Local professional norms or guidelines	^16^ ^,^ ^22^ ^–^ ^25^ ^,^ ^32^ ^,^ ^35^ ^,^ ^41^ ^,^ ^44^ ^,^ ^46^ ^,^ ^49^ ^,^ ^53^ ^,^ ^58^ ^,^ ^61^ ^,^ ^66^ ^,^ ^67^ ^,^ ^70^ ^–^ ^73^ ^,^ ^76^ ^,^ ^81^ ^–^ ^83^ ^,^ ^85^ ^,^ ^87^ ^,^ ^88^ ^,^ ^90^ ^,^ ^93^ ^–^ ^95^ ^,^ ^97^ ^,^ ^100^ ^,^ ^101^ ^,^ ^110^ ^,^ ^113^ ^,^ ^115^ ^,^ ^120^ ^,^ ^123^ ^–^ ^126^ ^,^ ^128^ ^,^ ^129^ ^,^ ^134^ ^,^ ^141^ ^,^ ^151^ ^–^ ^154^ ^,^ ^160^ ^,^ ^161^ ^,^ ^163^ ^,^ ^167^ ^,^ ^168^ ^,^ ^170^ ^,^ ^172^ ^,^ ^175^ ^,^ ^177^ ^–^ ^181^ ^,^ ^183^ ^,^ ^185^ ^,^ ^195^ ^,^ ^197^ ^,^ ^202^ ^,^ ^205^ ^,^ ^207^ ^,^ ^211^ ^,^ ^214^ ^,^ ^219^ ^,^ ^221^ ^,^ ^224^ ^,^ ^226^ ^,^ ^228^ ^,^ ^230^ ^,^ ^237^ ^,^ ^248^ ^,^ ^257^ ^,^ ^261^ ^,^ ^262^ ^,^ ^264^ ^,^ ^266^ ^,^ ^282^ ^,^ ^284^ ^,^ ^285^ ^,^ ^287^ ^–^ ^290^ ^,^ ^294^ ^,^ ^295^ ^,^ ^298^ ^,^ ^306^ ^,^ ^307^ ^,^ ^309^ ^,^ ^316^ ^,^ ^319^ ^,^ ^323^ ^,^ ^325^ ^,^ ^326^ ^,^ ^328^ ^,^ ^334^ ^,^ ^343^ ^,^ ^345^ ^,^ ^350^ ^,^ ^353^ ^,^ ^360^ ^,^ ^362^ ^,^ ^368^ ^,^ ^376^ ^,^ ^377^	114 (37.1)
Beers criteria® for Potentially Inappropriate Medication Use in Older Adults	^19^ ^,^ ^31^ ^,^ ^36^ ^,^ ^39^ ^,^ ^40^ ^,^ ^51^ ^,^ ^54^ ^,^ ^57^ ^,^ ^60^ ^,^ ^63^ ^,^ ^77^ ^–^ ^79^ ^,^ ^89^ ^,^ ^92^ ^,^ ^96^ ^,^ ^102^ ^,^ ^104^ ^–^ ^106^ ^,^ ^109^ ^,^ ^112^ ^,^ ^116^ ^,^ ^130^ ^,^ ^131^ ^,^ ^133^ ^,^ ^136^ ^–^ ^138^ ^,^ ^142^ ^,^ ^143^ ^,^ ^150^ ^,^ ^158^ ^–^ ^161^ ^,^ ^165^ ^,^ ^169^ ^,^ ^182^ ^,^ ^184^ ^,^ ^191^ ^,^ ^196^ ^,^ ^198^ ^,^ ^199^ ^,^ ^206^ ^,^ ^213^ ^,^ ^216^ ^,^ ^218^ ^,^ ^225^ ^,^ ^227^ ^,^ ^229^ ^,^ ^238^ ^,^ ^239^ ^,^ ^253^ ^,^ ^254^ ^,^ ^258^ ^,^ ^259^ ^,^ ^270^ ^,^ ^271^ ^,^ ^281^ ^,^ ^286^ ^,^ ^291^ ^,^ ^297^ ^,^ ^301^ ^,^ ^302^ ^,^ ^305^ ^,^ ^310^ ^–^ ^312^ ^,^ ^324^ ^,^ ^330^ ^,^ ^337^ ^,^ ^346^ ^,^ ^349^ ^,^ ^352^ ^,^ ^354^ ^,^ ^358^ ^,^ ^361^ ^,^ ^373^ ^,^ ^375^	80 (26.1)
Screening tool of older persons’ prescriptions	^20^ ^,^ ^33^ ^,^ ^34^ ^,^ ^59^ ^,^ ^60^ ^,^ ^78^ ^,^ ^102^ ^,^ ^107^ ^,^ ^130^ ^,^ ^132^ ^,^ ^133^ ^,^ ^142^ ^,^ ^146^ ^,^ ^161^ ^,^ ^184^ ^,^ ^188^ ^,^ ^196^ ^,^ ^199^ ^,^ ^204^ ^,^ ^217^ ^,^ ^218^ ^,^ ^225^ ^,^ ^229^ ^,^ ^243^ ^,^ ^250^ ^,^ ^253^ ^,^ ^254^ ^,^ ^258^ ^,^ ^311^ ^,^ ^317^ ^,^ ^337^ ^,^ ^352^	32 (10.4)

N/A: not applicable.

^a^ The denominator is all 367 studies included in the scoping review.

^b^ Including only single country studies.

^c^ Including anti-anxiety, antidepressants and antipsychotics.

Box 2 **Examples of key findings in the scoping review on overuse of medications in low- and middle-income countries**

**Estimation of overuse (307 studies; Table 2)**

For low-income countries (21 studies), the IQR of overuse estimates was 48.2–82.5%.

A cross-sectional study evaluating the appropriateness of antibiotic use for acute diarrhoeal disease among 303 inpatients in a large Ethiopian hospital serving 1.2 million people showed that 86.8% (263) of patients had received at least one antibiotic drug (92.6%; 174/188 of children younger than five years).^343^ The rate of overuse of antibiotics was 72.3%,^343^ which is a high level of overuse of antibiotics according to the recommendations of WHO for treating diarrhoeal disease.

For lower-middle-income countries (63 studies), the IQR of overuse estimates was 26.8–66.2%.

A retrospective analysis evaluating the use of potentially inappropriate medicines in 676 elderly patients in an Indian teaching hospital showed that 590 (87.3%) patients received at least one inappropriate medicine according to Beers criteria®, with metoclopramide being the most commonly used inappropriate medication (54.3%; 367 patients).^179^

For upper-middle-income countries (205 studies), the IQR of overuse estimates was 29.0–62.5.

A cross-sectional study evaluating the prevalence of potentially inappropriate medications among geriatric inpatients in a 3000-bed teaching hospital in China showed that the proportion of potentially inappropriate medications was 64.8% (4163/6424) according to Beers 2019 criteria® and 64.3% (4131/6424) according to Beers 2015 criteria®. Proton pump inhibitors were the most commonly prescribed inappropriate medications.^162^

**Drivers of overuse (139 studies; Table 3)**

A qualitative study explored the drivers of antibiotics overuse in Médecins Sans Frontières projects located in four low-income African countries. The study included 384 people who participated in semi-structured in-depth interviews, focus group discussions, and field observations. System-level drivers included failing health-care systems, such as underdeveloped and understaffed primary and secondary health-care systems. Individual-level drivers included strong patient demands for antibiotics that influenced prescription decisions, and high proportions of self-medication.^383^

Researchers of a qualitative study to identify drivers of potentially inappropriate medication use interviewed 417 geriatric patients in 22 public primary care facilities in Brazil, an upper-middle-income country. Important patient-related drivers included short consultation times (< 10 minutes) and polypharmacy. Clinician-related drivers included the number of patients attended and number of prescriptions per clinician.^43^

**Consequences of overuse (31 studies; Table 3)**

A cross-sectional study examined the costs and appropriateness of prophylactic preoperative antibiotic use among 1000 consecutive patients in six teaching hospitals in the Islamic Republic of Iran, an upper-middle-income country. Antibiotics were prescribed for 85 out of 87 (97.7%) procedures in which an antibiotic was not indicated. The average cost of antibiotic prescription per surgical procedure was US$ 100 and the estimated total cost of inappropriate prescription of cefazoline alone (the most frequently prescribed antibiotic) was US$ 4623; the GDP per capita is US$ 4802.^160^

A cross-sectional analysis was conducted of the economic impact of unnecessary proton pump inhibitors in Lebanon, an upper-middle-income country. The proportion of overuse was 71.4% (714/1000 individuals), with an estimated annual waste of US$ 25 million.^170^

**Solutions for the problem of overuse (42 studies; Table 3)**

A before-and-after study evaluated the effect of an intervention (training and pulse oximeters) on the appropriate use of oxygen among 1765 patients in a teaching hospital in Rwanda, a low-income country. Oxygen use in the emergency department decreased from a median of 32.0 (IQR: 28.0–35.0) tanks per day to 16.0 (IQR: 12.5–21.0) tanks per day at week 12. The proportion of appropriate use of oxygen therapy increased from 18.7% (34/182) at baseline to 42.0% (42/100) at 12 weeks.^329^

A multicentre RCT of the effect of point-of-care CRP testing on the inappropriate use of antibiotics for non-severe acute respiratory infections among 2037 patients in 10 Vietnamese primary health-care centres showed that the number of patients who used antibiotics within 14 days was 581 (64.4%) of 902 patients in the CRP group versus 738 (77.9%) of 947 patients in the control group (OR: 0.49; 95% CI: 0.40–0.61).^115^

CI: confidence interval; CRP: C-reactive protein; GDP: gross domestic product; IQR: interquartile range; OR: odds ratio; RCT: randomized controlled trial; US$: United States dollars; WHO: World Health Organization.Note: Results reported in the box are based on key studies that we have selected to represent different countries (i.e. income group or WHO region) and main theme or focus (e.g. solution or estimate). However, these studies were conducted in local settings and might not be representative to the wider low- and middle-income countries context. Therefore, generalizability of these findings to the low- and middle-income countries context is limited and should be done with extreme caution.

The top five classes of medications that were most frequently examined and reported in the 307 studies were (i) antimicrobials (110 studies; 35.8%); (ii) anti-anxiety, antidepressants and antipsychotics (89 studies; 30.0%); (iii) antihypertensive drugs (74 studies; 24.1% ); (iv) analgesics and steroids (67 studies; 21.8%); and (v) proton pump inhibitors and antacids (48 studies; 15.6%; Table 2).

The three most common methods for assessing overuse of medications were: comparing existing prescribing practices with what would be considered appropriate under local professional norms or guidelines (114 studies; 37.1%); using Beers criteria® to identify potentially inappropriate medications (80 studies; 26.1%); and using STOPP (32 studies; 10.4%; Table 2).

#### Antimicrobials

The estimate of the overuse of antibiotics in the 110 studies range from 18.4% (40/217 prescriptions) in a retrospective evaluation of the inappropriateness of antibiotics prescribed for 735 patients in three primary health care clinics in Malaysia^309^ to 97.0% (194) of 200 pharmacies inappropriately dispensing antibiotics without prescriptions in the Syrian Arab Republic.^36^ A 2019 Chinese survey of more than 74 648 antibiotics prescriptions in 16 rural primary care health centres found that only 8.8% (6567) of prescriptions were appropriate and 84.1% (62 780) of prescriptions were deemed unnecessary, according to guidelines.^96^ A 2021 systematic review of 23 studies estimated the proportion of nonprescription inappropriate dispensing of antibiotics in sub-Saharan African countries at 69.0% (95% confidence interval, CI: 58.0–80.0).^73^ The IQR of the estimates reported in the 110 studies was 34.3–66.5%. In the 14 low-income countries included, the estimates ranged between 53.9% and 85.4%; the ranges were 30.5–64.3% and 34.3–60.9% for 28 lower-middle- and 61 upper-middle-income countries, respectively (Table 2).

#### Psychotropic drugs

The estimates of the overuse of anti-anxiety and antidepressant medications in the 89 studies range from 10.0 % in a survey of 1048 Chilean adults 65 years or older according to Beers criteria®^269^ to 91.0 % in a cross-sectional analysis of 456 older adults in a tertiary hospital in India using both Beers® and STOPP criteria.^311^ The IQR of the estimates reported in the 89 studies was 30.1–61.7%. There was no study for a low-income country. For 10 lower-middle- and 77 upper-middle-income countries, the estimate ranges were 28.6–87.3% and 31.2–61.4%, respectively (Table 2).

#### Analgesics, proton pump inhibitors and antihypertensive drugs

A cross-sectional analysis of medical records of 13 274 elderly patients in four community hospitals in Thailand found that 79.0% (10486) of patients were prescribed one or more potentially inappropriate medications, using the Lists of Risk Drugs for Thai Elderly criteria, with antihypertensive drugs, proton pump inhibitors and analgesics being the most frequently prescribed potentially inappropriate medications.^274^ Another cross-sectional analysis of medical records of 6337 older adults in China found 79.4% (5033) prescribed one or more potentially inappropriate medications according to either Beers criteria® or STOPP, with antihypertensive, anti-anxiety and non-steroidal anti-inflammatory drugs among the most common potentially inappropriate medications.^201^

### Drivers

Drivers and factors related to the overuse of medications in low- and middle-income countries were reported in 139 (37.9%) studies, with 74 studies reporting on individual-level (i.e. clinician or patient, e.g. knowledge and skills or preferences) and 40 on system-level drivers or factors (i.e. institutional or organization, e.g. resource allocation, staffing and national guidelines; Table 3). Only 10 of these studies were from low-income countries,^20^^,^^77^^,^^111^^,^^127^^,^^129^^,^^144^^,^^166^^,^^183^^,^^262^^,^^337^ and we did not observe clear associations between country income levels and drivers (Box 2), nor could we observe any geographical variations in the drivers.

**Table 3:** Studies included on the drivers, solutions and consequences of medication overuse in low- and middle-income countries

Study characteristic	Study reference	No. (%) of studies
**Drivers**	^15^ ^,^ ^18^ ^,^ ^20^ ^,^ ^27^ ^–^ ^30^ ^,^ ^32^ ^,^ ^36^ ^,^ ^40^ ^,^ ^43^ ^,^ ^44^ ^,^ ^47^ ^,^ ^48^ ^,^ ^52^ ^–^ ^54^ ^,^ ^60^ ^,^ ^63^ ^,^ ^68^ ^,^ ^69^ ^,^ ^71^ ^,^ ^77^ ^,^ ^79^ ^,^ ^82^ ^,^ ^86^ ^,^ ^88^ ^–^ ^92^ ^,^ ^96^ ^,^ ^99^ ^,^ ^102^ ^,^ ^104^ ^,^ ^105^ ^,^ ^111^ ^,^ ^113^ ^,^ ^115^ ^,^ ^118^ ^,^ ^119^ ^,^ ^121^ ^,^ ^127^ ^,^ ^129^ ^,^ ^130^ ^,^ ^132^ ^,^ ^135^ ^–^ ^139^ ^,^ ^144^ ^,^ ^151^ ^,^ ^153^ ^,^ ^155^ ^,^ ^156^ ^,^ ^159^ ^,^ ^162^ ^,^ ^164^ ^–^ ^166^ ^,^ ^173^ ^–^ ^175^ ^,^ ^183^ ^,^ ^190^ ^,^ ^193^ ^,^ ^194^ ^,^ ^196^ ^,^ ^198^ ^,^ ^199^ ^,^ ^204^ ^,^ ^206^ ^,^ ^208^ ^,^ ^210^ ^,^ ^213^ ^,^ ^216^ ^–^ ^218^ ^,^ ^221^ ^,^ ^222^ ^,^ ^226^ ^,^ ^228^ ^,^ ^229^ ^,^ ^234^ ^,^ ^235^ ^,^ ^238^ ^,^ ^239^ ^,^ ^244^ ^,^ ^246^ ^,^ ^247^ ^,^ ^250^ ^–^ ^252^ ^,^ ^260^ ^,^ ^262^ ^,^ ^275^ ^,^ ^277^ ^,^ ^278^ ^,^ ^283^ ^,^ ^284^ ^,^ ^286^ ^,^ ^291^ ^,^ ^292^ ^,^ ^295^ ^,^ ^297^ ^,^ ^300^ ^,^ ^302^ ^–^ ^304^ ^,^ ^307^ ^,^ ^309^ ^,^ ^310^ ^,^ ^312^ ^,^ ^317^ ^,^ ^319^ ^,^ ^321^ ^,^ ^322^ ^,^ ^326^ ^,^ ^327^ ^,^ ^332^ ^,^ ^333^ ^,^ ^337^ ^–^ ^340^ ^,^ ^342^ ^,^ ^346^ ^,^ ^350^ ^,^ ^352^ ^,^ ^354^ ^,^ ^357^ ^,^ ^358^ ^,^ ^362^ ^,^ ^364^ ^,^ ^366^ ^,^ ^368^ ^,^ ^371^ ^,^ ^379^	139 (37.9)^a^
Individual-level factors	^27^ ^,^ ^29^ ^,^ ^40^ ^,^ ^43^ ^,^ ^47^ ^,^ ^48^ ^,^ ^53^ ^,^ ^60^ ^,^ ^63^ ^,^ ^69^ ^,^ ^71^ ^,^ ^77^ ^,^ ^79^ ^,^ ^82^ ^,^ ^86^ ^,^ ^88^ ^–^ ^90^ ^,^ ^92^ ^,^ ^96^ ^,^ ^99^ ^,^ ^102^ ^,^ ^104^ ^,^ ^105^ ^,^ ^118^ ^,^ ^119^ ^,^ ^121^ ^,^ ^127^ ^,^ ^130^ ^,^ ^136^ ^–^ ^139^ ^,^ ^144^ ^,^ ^151^ ^,^ ^153^ ^,^ ^155^ ^,^ ^156^ ^,^ ^159^ ^,^ ^165^ ^,^ ^166^ ^,^ ^173^ ^–^ ^175^ ^,^ ^190^ ^,^ ^193^ ^,^ ^196^ ^,^ ^208^ ^,^ ^210^ ^,^ ^213^ ^,^ ^229^ ^,^ ^238^ ^,^ ^247^ ^,^ ^250^ ^,^ ^251^ ^,^ ^260^ ^,^ ^283^ ^,^ ^286^ ^,^ ^291^ ^,^ ^300^ ^,^ ^302^ ^–^ ^304^ ^,^ ^310^ ^,^ ^312^ ^,^ ^332^ ^,^ ^337^ ^,^ ^339^ ^,^ ^354^ ^,^ ^357^ ^,^ ^358^ ^,^ ^366^ ^,^ ^371^ ^,^ ^379^	74 (53.2)
Polypharmacy and/or multimorbidity	^27^ ^,^ ^40^ ^,^ ^60^ ^,^ ^63^ ^,^ ^77^ ^,^ ^79^ ^,^ ^86^ ^,^ ^88^ ^–^ ^90^ ^,^ ^92^ ^,^ ^96^ ^,^ ^102^ ^,^ ^104^ ^,^ ^105^ ^,^ ^130^ ^,^ ^136^ ^–^ ^139^ ^,^ ^159^ ^,^ ^165^ ^,^ ^174^ ^,^ ^196^ ^,^ ^208^ ^,^ ^213^ ^,^ ^229^ ^,^ ^250^ ^,^ ^286^ ^,^ ^291^ ^,^ ^300^ ^,^ ^303^ ^,^ ^304^ ^,^ ^310^ ^,^ ^337^ ^,^ ^354^ ^,^ ^358^ ^,^ ^379^	38 (51.4)
Limited knowledge	^29^ ^,^ ^47^ ^,^ ^48^ ^,^ ^69^ ^,^ ^82^ ^,^ ^99^ ^,^ ^118^ ^,^ ^119^ ^,^ ^121^ ^,^ ^127^ ^,^ ^144^ ^,^ ^166^ ^,^ ^175^ ^,^ ^190^ ^,^ ^210^ ^,^ ^238^ ^,^ ^250^ ^,^ ^251^ ^,^ ^283^ ^,^ ^302^ ^,^ ^312^ ^,^ ^332^ ^,^ ^357^ ^,^ ^371^	24 (32.4)
Patient demand	^43^ ^,^ ^47^ ^,^ ^48^ ^,^ ^53^ ^,^ ^71^ ^,^ ^193^ ^,^ ^210^ ^,^ ^247^ ^,^ ^260^ ^,^ ^339^ ^,^ ^366^ ^,^ ^371^	12 (16.2)
System-level factors	^15^ ^,^ ^18^ ^,^ ^28^ ^,^ ^40^ ^,^ ^43^ ^,^ ^47^ ^,^ ^48^ ^,^ ^52^ ^,^ ^68^ ^,^ ^69^ ^,^ ^71^ ^,^ ^89^ ^,^ ^92^ ^,^ ^99^ ^,^ ^111^ ^,^ ^113^ ^,^ ^119^ ^,^ ^127^ ^,^ ^130^ ^,^ ^139^ ^,^ ^151^ ^,^ ^156^ ^,^ ^164^ ^,^ ^166^ ^,^ ^190^ ^,^ ^193^ ^,^ ^196^ ^,^ ^210^ ^,^ ^213^ ^,^ ^246^ ^,^ ^247^ ^,^ ^251^ ^,^ ^260^ ^,^ ^283^ ^,^ ^310^ ^,^ ^332^ ^,^ ^333^ ^,^ ^357^ ^,^ ^366^ ^,^ ^371^	40 (28.8)
Constrained resources	^18^ ^,^ ^28^ ^,^ ^48^ ^,^ ^68^ ^,^ ^69^ ^,^ ^71^ ^,^ ^111^ ^,^ ^139^ ^,^ ^166^ ^,^ ^190^ ^,^ ^210^ ^,^ ^247^ ^,^ ^251^ ^,^ ^260^ ^,^ ^333^ ^,^ ^357^	16 (40.0)
Financial factors	^15^ ^,^ ^47^ ^,^ ^48^ ^,^ ^119^ ^,^ ^164^ ^,^ ^166^ ^,^ ^193^ ^,^ ^210^ ^,^ ^247^ ^,^ ^251^ ^,^ ^283^ ^,^ ^366^ ^,^ ^371^	13 (32.5)
Regulatory issues	^15^ ^,^ ^43^ ^,^ ^48^ ^,^ ^69^ ^,^ ^99^ ^,^ ^119^ ^,^ ^127^ ^,^ ^193^ ^,^ ^251^ ^,^ ^332^ ^,^ ^371^	11 (27.5)
Other	^18^ ^,^ ^40^ ^,^ ^52^ ^,^ ^89^ ^,^ ^92^ ^,^ ^113^ ^,^ ^130^ ^,^ ^151^ ^,^ ^156^ ^,^ ^196^ ^,^ ^213^ ^,^ ^246^ ^,^ ^310^	13 (32.5)
**Consequences**	^21^ ^,^ ^23^ ^,^ ^33^ ^–^ ^35^ ^,^ ^37^ ^,^ ^39^ ^,^ ^46^ ^,^ ^74^ ^,^ ^81^ ^,^ ^103^ ^,^ ^112^ ^,^ ^116^ ^,^ ^142^ ^,^ ^143^ ^,^ ^179^ ^,^ ^203^ ^,^ ^242^ ^,^ ^265^ ^,^ ^268^ ^,^ ^274^ ^,^ ^280^ ^,^ ^294^ ^,^ ^308^ ^,^ ^315^ ^,^ ^318^ ^,^ ^331^ ^,^ ^361^ ^,^ ^365^ ^,^ ^374^ ^,^ ^380^	31 (8.4)^a^
**Potential solutions**	^17^ ^,^ ^25^ ^,^ ^43^ ^,^ ^48^ ^,^ ^55^ ^,^ ^64^ ^,^ ^66^ ^,^ ^98^ ^,^ ^99^ ^,^ ^106^ ^,^ ^114^ ^,^ ^117^ ^,^ ^141^ ^,^ ^147^ ^,^ ^170^ ^,^ ^176^ ^,^ ^186^ ^,^ ^189^ ^,^ ^193^ ^,^ ^200^ ^,^ ^201^ ^,^ ^205^ ^,^ ^209^ ^,^ ^215^ ^,^ ^231^ ^,^ ^236^ ^,^ ^240^ ^,^ ^249^ ^,^ ^274^ ^,^ ^276^ ^,^ ^299^ ^,^ ^305^ ^,^ ^313^ ^,^ ^329^ ^,^ ^335^ ^,^ ^341^ ^,^ ^347^ ^,^ ^356^ ^,^ ^359^ ^,^ ^363^ ^,^ ^378^ ^,^ ^381^	42 (11.4)^a^
Study design		
Interventional	^55^ ^,^ ^66^ ^,^ ^98^ ^,^ ^106^ ^,^ ^114^ ^,^ ^117^ ^,^ ^176^ ^,^ ^186^ ^,^ ^189^ ^,^ ^201^ ^,^ ^215^ ^,^ ^231^ ^,^ ^236^ ^,^ ^240^ ^,^ ^249^ ^,^ ^299^ ^,^ ^313^ ^,^ ^329^ ^,^ ^341^ ^,^ ^347^ ^,^ ^356^ ^,^ ^359^ ^,^ ^363^	23 (54.8)
Observational	^17^ ^,^ ^25^ ^,^ ^43^ ^,^ ^48^ ^,^ ^64^ ^,^ ^99^ ^,^ ^141^ ^,^ ^147^ ^,^ ^170^ ^,^ ^193^ ^,^ ^200^ ^,^ ^205^ ^,^ ^209^ ^,^ ^274^ ^,^ ^276^ ^,^ ^305^ ^,^ ^335^ ^,^ ^378^ ^,^ ^381^	19 (45.2)
Medicine focus		
Antibiotics	^17^ ^,^ ^25^ ^,^ ^48^ ^,^ ^55^ ^,^ ^64^ ^,^ ^98^ ^,^ ^99^ ^,^ ^114^ ^,^ ^117^ ^,^ ^141^ ^,^ ^147^ ^,^ ^205^ ^,^ ^209^ ^,^ ^231^ ^,^ ^236^ ^,^ ^249^ ^,^ ^274^ ^,^ ^299^ ^,^ ^313^ ^,^ ^347^ ^,^ ^363^ ^,^ ^381^	22 (52.4)
Others	^43^ ^,^ ^66^ ^,^ ^106^ ^,^ ^170^ ^,^ ^176^ ^,^ ^186^ ^,^ ^189^ ^,^ ^193^ ^,^ ^200^ ^,^ ^201^ ^,^ ^215^ ^,^ ^240^ ^,^ ^276^ ^,^ ^305^ ^,^ ^329^ ^,^ ^335^ ^,^ ^341^ ^,^ ^356^ ^,^ ^359^ ^,^ ^378^	20 (47.6)

^a^ The denominator is all 367 studies included in the scoping review.

#### Individual-level drivers

Of the studies reporting on individual-level drivers, 24 studies cited limited knowledge among clinicians or patients on the harms of overuse, 12 studies pointed to perceived or actual patients’ demands, while polypharmacy and/or multimorbidity was found as a factor in 38 studies, largely studies of potentially inappropriate medications among elderly people (Table 3). For example, a study of 6400 hospitalized older patients in China found that 64.8% (4147) of prescriptions were inappropriate based on Beers criteria®, with proton pump inhibitors the most common potentially inappropriate medication, and polypharmacy was the most important factor associated with potentially inappropriate medications.^162^ In a qualitative study with clinicians and older adults in Burkina Faso, a low-income country, drivers of overuse included poor patient–clinician communication, limited clinician knowledge, and incentives from pharmaceutical companies.^163^

#### System-level drivers

Of the studies reporting on system-level drivers and factors, 11 studies cited regulatory issues such as poorly enforced regulations or policies; 16 studies cited constrained resources, including limited access to diagnostic facilities, and problematic access in rural or regional places; and 13 studies cited financial factors such as profitability of health-care providers and industry influence (Table 3). For example, a nationwide Malaysian study of 5810 antibiotic prescriptions in 545 clinics (129 public and 416 private clinics) found much higher proportions of antibiotic prescriptions (30.8%; 5055/16 415 vs 6.8%; 755/11 172), and antibiotic prescriptions for respiratory tract infections (16.7%; 49/293 vs 57.7%; 1479/2564) in private clinics versus public, most of which were considered unnecessary.^15^

### Consequences

Consequences of overuse of medications were reported in 31 studies (8.4%), including just one from a low-income country (Table 3).^21^ The two most commonly identified consequences of overuse were harms – such as adverse drug reactions, impacts on quality of life or mortality – and costs. For example, a study among 125 heart-failure patients in Lebanon found a moderate correlation between potentially inappropriate medications and reduced quality of life,^374^ while another small study of 127 older adults in Brazil found no impact of potentially inappropriate medications on mortality, during a 10-year follow-up.^111^ In relation to costs, a study in the Islamic Republic of Iran estimated that almost 1 million United States dollars (US$) was wasted within a year in one teaching hospital on unnecessary antibiotics, measured as use not adherent to local guidelines.^292^ In 2020, a survey in Lebanon found widespread overuse of proton pump inhibitors and estimated that the nation may be wasting US$ 25 million annually as a result.^170^ No studies in low-income countries assessed costs of overuse.

### Potential solutions

Evaluations of potential solutions were reported in 42 studies (11.4%), with 23 studies reporting on interventional rather than observational studies (Table 3). Only four studies of solutions were based in low-income countries^231^^,^^249^^,^^329^^,^^335^ (data repository).^14^

Twenty-two studies of solutions focused on antibiotics (Table 3). For example, a review identified multiple interventions that successfully reduced inappropriate antibiotics in low- and middle-income countries, including national action plans, educational campaigns, wider use of audit–feedback initiatives and regulatory or legal changes to reduce self-purchasing of antibiotics.^145^ Similarly, a large study of long-term antibiotic use in 55 low- and middle-income countries, using World Health Organization databases, found that having national policies and/or strategies on rational antibiotic use was associated with an estimated 20% reduction in antibiotic use for upper respiratory tract infections and an estimated 30% reduction in antibiotic use for acute diarrhoeal illness.^167^

For non-antibiotic overuse, 20 studies evaluated potential solutions, including educational interventions, deprescribing initiatives, new technologies and interventions involving pharmacists (Table 3). In Uganda, a low-income country, two cluster randomized controlled trials of 127 villages and 381 community health workers found dramatic reductions in the overuse of antimalarial drugs following the introduction of rapid diagnostic tests (48.5% absolute reduction: from 79.3%; 520/656; to 30.8%; 215/699).^247^^,^^388^ In Argentina, a multifaceted intervention including educational workshops, deprescribing algorithms and automated email alerts was found to successfully reduce inappropriate medications among 900 older adults (overall absolute reduction of 8.5% across eight classes of potentially inappropriate medications – relative reduction of 73% for non-steroidal anti-inflammatory drugs and 31% for benzodiazepines).^303^ Also in Argentina, an email-linked behavioural intervention underpinned by social-norm feedback reduced inappropriate prescriptions and cost for cognitively impaired older adults. Physicians in the intervention group made fewer inappropriate prescriptions than physicians in the control group, mean 93.25 prescriptions (95% CI: 89.27–97.24) versus 98.99 (95% CI: 95.00–102.98).^341^ In Thailand, a large study of 11 915 patients across four community hospitals found that computerized decision support systems that detect potentially inappropriate medications were associated with 13.3 percentage points decrease (from 87.7% to 74.4%) in potentially inappropriate medications.^275^

A 2021 Chinese study found that an intervention by clinical pharmacists halved the rate of inappropriate prescriptions of proton pump inhibitors (from 48.9 to 22.7 prescriptions per 100 patient-days), with no harmful impacts.^378^ Encouragingly, a 2018 survey in Ethiopia, a low-income country, assessing attitudes to deprescribing, found that 81.6% (258/316) of people asked were willing to stop one or more medications if possible and if agreed by their doctors.^335^

## Discussion

We found widespread evidence of high proportions of overuse of medications in low- and middle-income countries. Classes of commonly overused medications included antibiotics, benzodiazepines, non-steroidal anti-inflammatory drugs, proton pump inhibitors and antihypertensive medicines. Drivers of overuse included a lack of knowledge of overuse among patients or clinicians, insufficient resources to access optimum care, polypharmacy, financial factors including the profit motive, and weak or poorly enforced regulations. Consequences were harms from adverse drug reactions and high costs of inappropriate use of scarce resources. While only a small number of studies evaluated potential solutions, we identified feasible and effective interventions, even for the lowest income settings, to tackle the problem of overuse.

Almost 90% of the included studies were published in the past 10 years, emphasizing the growing interest in understanding and addressing overuse of medications in low- and middle-income countries. We observed wide dispersions of the proportion of overuse of medications; this finding might be attributed to the differences in the population, settings, medications and methods used among included studies. Only a few studies evaluated the consequences, such as financial wasting. Wasting of resources is catastrophic for health-care systems in low- and middle-income countries that are already fragmented and fragile.^389^ Future research should not only estimate the extent of overuse of prescribed medications, but also estimate the extent and drivers of the overuse from nonprescription medications. Furthermore, studies should evaluate the potential physical, psychological and social consequences, both at individual and health-care system levels. Further research should also include improving the methods to evaluate the harms and consequences associated with overuse of medications.

The finding that only a tenth of studies evaluated potential solutions points to a gap in the literature and the need to intensify research evaluating innovative solutions to reduce overuse, while at the same time improving the rigor of the science assessing the extent and nature of the problem. Our review finds evidence from low- and middle-income countries of significant reductions in inappropriate medications, using for example multifaceted interventions or deprescribing initiatives, which suggests such interventions are practical as well as desirable. However, future studies replicating successful interventions from high-income countries need to include more robust evaluations of individual-level (e.g. educational interventions, audit and feedback, and clinical decision support system) and system-level solutions (e.g. changes in payment and incentives and changes in regulations and policies).^390^

We also found strong evidence of very high levels of overprescription of antibiotics across many low- and middle-income countries. Such overprescription is associated with antibiotic resistance,^391^ however, there are hopeful signs that those overprescription rates can be reduced with national policies and local initiatives.^145^^,^^167^ Research evaluating potential solutions for unnecessary prescription needs to focus on nonprescription antibiotic dispensing in drug shops and antibiotic prescriptions by private health-care providers. This research might include multicomponent interventions combining educational programmes to improve public awareness; engagement of pharmacists in patient education and consumer guideline development; audit and feedback; and more incisive regulations and policies to reduce inappropriate antibiotic use in low- and middle-income countries.^392^

Our finding that only 7% of studies were from low-income countries suggests that either the problem of overuse may not be so pressing in places where underuse is a clear priority for stakeholders, or that the problem of overuse is understudied.

Our review has limitations. To achieve the breadth required of a scoping review, we have necessarily included studies using a range of definitions and methods, from assessing adherence to guidelines for appropriate use, to the potentially inappropriate medications assessments using the Beers criteria®, STOPP and other criteria. While we broadly accepted the definitions used by authors of the studies, if our review team could find no evidence in the study of an explicit method to measure overuse, the study was excluded. Generally, definitions of inappropriate use equate to use when a medication is not clinically indicated, or the overuse of a medication which may bring more harm than benefit, but in some studies, use of the term inappropriate also included finer details relating to dosage, frequency or duration of use. A further limitation arises from our subjective categorization of system- versus individual-level drivers, where there may be clear overlap among these drivers. Another minor limitation arises from applying the World Bank income level list from 2021 to studies conducted over many previous years, however, the income level has not changed for most low- and middle-income countries.^13^

The findings of this review suggest the time has arrived to build a global community of researchers, clinicians, policy-makers and citizens who will share, build and improve the science of overuse in low- and middle-income countries. The global community should have a focus on scaling up effective solutions to reduce harm and save wasted resources. Initiatives to tackle overuse of medications in low- and middle-income countries are currently limited and of a particular focus – such as the Global Antimicrobial Resistance Partnership.^393^^–^^395^ This review’s broad findings confirm the need for global discussions to prioritize research and action agendas. Global initiatives which have largely arisen in high-income countries to tackle overuse, such as Right Care, Preventing Overdiagnosis and Choosing Wisely, will only be strengthened through developing links with, and learning from, colleagues in low- and middle-income countries.
